# Synergistic Phytochemicals From *Mitracarpus frigidus* (Willd. ex Roem. & Schult.) K. Schum. and *Lantana camara* Linn. in Chitosan Biofilms: Antioxidant and Anti‐Inflammatory Activities With Wound‐Healing Potential

**DOI:** 10.1002/cbdv.71645

**Published:** 2026-09-30

**Authors:** Natasha Silva Mayrink, Julia Bertolini Fajardo, Mariane Rocha Cordeiro Comitre, Lucas de Araújo Carvalho, Mariana Iasbeck Dalcol, Priscila de Lima Paula, Lara Melo Campos, Maria Clara Machado Resende Guedes, Luiz Fernando Cappa de Oliveira, Elaine Soares Coimbra, Gilson Costa Macedo, Ângelo Márcio Leite Denadai, Ari Sérgio de Oliveira Lemos, Rodrigo Luiz Fabri

**Affiliations:** ^1^ Department of Biochemistry Laboratory of Bioactive Natural Products Institute of Biological Sciences Federal University of Juiz de Fora Juiz de Fora Minas Gerais Brazil; ^2^ Department of Parasitology Microbiology and Immunology Institute of Biological Sciences Federal University of Juiz de Fora Juiz de Fora Minas Gerais Brazil; ^3^ Department of Pharmacy Institute of Life Sciences Federal University of Juiz de Fora Governador Valadares Minas Gerais Brazil

**Keywords:** adhesive films, biological activity, inflammation, multi‐functional, skin disorders

## Abstract

Skin disorders associated with inflammation and oxidative stress remain challenging to treat, highlighting the need for safe and effective topical therapies. This study investigated the association of aqueous extract of *Mitracarpus frigidus* aerial parts (MFAq) and hydroalcoholic extract of *Lantana camara* leaves (LCHA) incorporated into chitosan‐based films for potential dermatological applications. Different extract ratios were initially evaluated for phytochemical composition, cytotoxicity, antioxidant, and anti‐inflammatory activities. The 1:1 MFAq:LCHA association was selected based on its balanced phytochemical profile, low cytotoxicity, and significant biological activity, with eleven major compounds identified by UFLC‐QTOF‐MS. This association reduced nitric oxide production by 88.83% and lipid droplet accumulation by 59.48%, while showing pronounced antioxidant activity. Chitosan films containing 0.5%, 1.0%, and 2.5% (w/w) of the selected association were homogeneous, thermally stable, and exhibited suitable mechanical and physicochemical properties for topical application. The films maintained high cell viability and demonstrated significant antioxidant, anti‐inflammatory, and wound‐healing activities, including reductions in nitric oxide (up to 59.58%), reactive oxygen species, and pro‐inflammatory cytokines (up to 40.57% and 62.27% for IL‐6 and IL‐12), as well as enhanced fibroblast migration. Overall, these findings indicate that MFAq:LCHA‐loaded chitosan films represent a promising biodegradable and multifunctional platform for the treatment of skin disorders associated with inflammation and oxidative stress. Further in vivo studies are warranted to confirm their therapeutic efficacy and safety.

## Graphical abstract

Biodegradable chitosan films loaded with a 1:1 combination of *Mitracarpus frigidus* and *Lantana camara* extracts demonstrated potent antioxidant, anti‐inflammatory, and wound‐healing properties, while exhibiting low cytotoxicity and favorable physicochemical characteristics. These multifunctional films show great promise as topical treatments for inflammatory and oxidative stress‐related skin disorders.

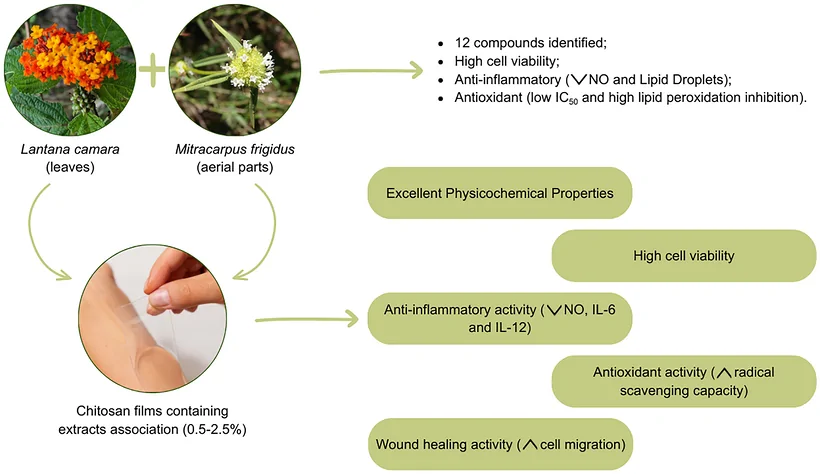

## 1 Introduction

The skin, the largest organ in the human body, has as its main functions of protection and thermoregulation. However, with aging and exposure to environmental factors, the integrity and protective function of the skin can become compromised. In addition, several skin disorders associated with inflammation and oxidative stress remain challenging to treat effectively, which highlights the need for alternatives for treating these conditions [1]. Oxidative stress occurs when there is an intense inflammatory response, leading to increased production of reactive oxygen species (ROS), released by activated inflammatory cells. These species cause tissue destruction and DNA damage. When the body detects ROS, it triggers an inflammatory process as an immune response to harmful agents, aiming to eliminate pathogens, damaged cells, and toxins, promoting tissue recovery [2].

In this context, the use of topical formulations containing natural products has attracted increasing attention due to their versatility, enabling multifunctional therapeutic applications [3]. The search for effective and safe alternatives for treatment of inflammatory skin diseases is an active area of research, and the use of biopolymers and plant extracts represents a promising strategy.

Chitosan is considered an ideal material for topical formulations due to its biodegradability, biocompatibility, non‐toxicity, bioadhesive properties, biological activity, and hemostatic effect [4]. It is obtained through the partial or complete deacetylation of chitin, the second most abundant biopolymer in nature. Consequently, the market has increasingly focused on chitosan due to its high biological potential, particularly when combined with natural extracts, which can synergistically enhance its therapeutic benefits [5]. Chitosan‐based films have attracted increasing attention in the health field due to their multifunctional properties and effectiveness in treating various dermatological conditions, representing a promising alternative to conventional anti‐inflammatory agents [6].

*Lantana camara* Linn. and *Mitracarpus frigidus* (Willd. ex Roem. & Schult.) K. Schum. are plant species widely recognized in traditional medicine for their rich phytochemical profiles and diverse biological activities*. L. camara*, prevalent in tropical regions, is traditionally used as a diuretic, expectorant, febrifuge, and anti‐rheumatic agent [7]. Phytochemical investigations have demonstrated the presence of several bioactive metabolites, including flavonoids, iridoids, triterpenes, phenolic compounds, and glycosylated derivatives [8, 9]. In particular, UFLC‐QTOF‐MS analyses of the hydroalcoholic leaf extract (LCHA) revealed compounds such as lamiide, geniposide, pectolinarin, luteolin‐7‐O‐glucoside, and rhamnocitrin‐O‐glucoside. Among these, luteolin‐7‐O‐glucoside has been previously reported as one of the major constituents of *L. camara* leaves and is associated with remarkable antioxidant and anti‐inflammatory properties. Additionally, iridoid glycosides such as geniposide and terpene glycosides such as lamiide are known for their anti‐inflammatory and protective biological effects. Our research group previously demonstrated significant antioxidant and anti‐inflammatory activities of LCHA [7].

Similarly, species of the genus *Mitracarpus* are widely employed in traditional medicine for various ailments, including topical applications for skin conditions [10]. *Mitracarpus frigidus*, in particular, exhibits promising antioxidant and antimicrobial activities, suggesting its utility in pharmaceutical applications [2]. Its chemical profile is characterized by a predominance of alkaloids, triterpenes, and phenolic compounds, alongside flavonoids, steroids, and terpenes in extracts from leaves and aerial parts [2, 11]. Chemical characterization of the aqueous extract of aerial parts (MFAq) by UFLC‐QTOF‐MS identified several bioactive constituents, including chlorogenic acid, harounoside, clarinoside, quercetin, rutin, kaempferol, and 2‐azaanthraquinone, in addition to sucrose as a major soluble carbohydrate component. Many of these compounds, particularly chlorogenic acid, quercetin, rutin, and kaempferol, are extensively reported in the literature for their antioxidant, anti‐inflammatory, antimicrobial, and wound‐healing properties. Previous studies conducted by our research group also demonstrated promising effects of MFAq against oxidative stress and inflammatory processes [2].

Considering the promising antioxidant and anti‐inflammatory activities previously reported for LCHA and MFAq, the combination of these extracts represents a rational strategy to broaden the spectrum of bioactive compounds and potentially promote synergistic effects. Such an approach may enhance therapeutic efficacy while reducing the concentration required for each individual extract. However, although both extracts have been individually investigated, no previous studies have evaluated their association, the biological effects of different LCHA:MFAq ratios, or the incorporation of this combination into chitosan‐based films for topical applications. Thus, this study aimed to chemically and biologically characterize LCHA:MFAq associations at different proportions. The most promising ratio was subsequently incorporated into chitosan‐based films, which were physicochemically characterized and evaluated in vitro for antioxidant, anti‐inflammatory, and wound‐healing activities as a potential multifunctional formulation for the treatment of skin disorders associated with inflammation and oxidative stress.

## 2 Results and Discussion

### 2.1 Evaluation of MFAq:LCHA Associations

#### 2.1.1 Chemical Composition

To achieve a deeper understanding of the therapeutic properties and chemical characteristics of the MFAq:LCHA associations, phenolic compounds and terpenoids were quantified. Phenolics are well recognized as more polar, whereas the polarity of terpenoids depends on the functional groups present in their structures. This quantification of metabolites aimed to provide a comprehensive overview of the chemical profile of the developed associations.

As illustrated in Table 1, the content of phenolic and terpenoid compounds in MFAq:LCHA associations varies with different ratios. The phenolic content, expressed as tannic acid equivalents (µg/mg), was 76.80 ± 3.42 for the 1:1 ratio, 85.79 ± 13.26 for 4:1, 73.01 ± 2.84 for 3:2, 61.88 ± 12.76 for 2:3, and 57.38 ± 4.60 for 1:4. Conversely, terpenoid levels, expressed as lupeol equivalents (µg/mg), were 98.19 ± 0.29 for 1:1, 89.03 ± 2.82 for 4:1, 102.77 ± 2.99 for 3:2, 86.59 ± 2.40 for 2:3, and 105.78 ± 6.96 for 1:4.

**TABLE 1:** Determination of phenolic and terpenoid constituents in the five MFAq:LCHA association ratios.

Associations (ratios)	Phenols (µg/mg expressed as tannic acid equivalents)	Terpenes (µg/mg expressed as lupeol equivalents)
4:1	85.79 ± 13.26^d^, ^e^	89.03 ± 2.82^d^
3:2	73.01 ± 2.84^e^	102.77 ± 2.99^d^
1:1	76.80 ± 3.42^c^	98.19 ± 0.29^d^
2:3	61.88 ± 12.76^a^	86.59 ± 2.40^a^, ^b^, ^c^, ^e^
1:4	57.38 ± 4.60^a^, ^b^, ^c^	105.78 ± 6.96^d^

*Note*: Data are presented as mean ± standard deviation (SD) of three independent experiments performed in triplicate (*n* = 9). Statistical analysis was performed using one‐way ANOVA followed by Bonferroni's post hoc test. Differences were considered statistically significant at *p* < 0.05. Different letters indicate statistically significant differences between groups:

^a^ different from 4:1;

^b^ different from 3:2;

^c^ different from 1:1;

^d^ different from 2:3;

^e^ different from 1:4.

A clear trend emerged showing that increasing the proportion of MFAq in the mixture resulted in higher phenolic content. On the other hand, the lowest phenolic levels were observed with lower MFAq ratios (2:3 and 1:4). These results support the hypothesis that MFAq inherently contains a higher concentration of phenolic compounds than LCHA does, which significantly contributes to the overall phenolic profile of the combinations. In contrast, terpenoid content remained consistent across the associations, except for the 2:3 ratio (*p <* 0.05), which exhibited the lowest concentration. Therefore, the results indicate variations in the content of phenolic compounds between 5.73% and 8.57%, as well as variations in the content of terpenoids between 8.65% and 10.57%.

These findings are consistent with previous studies reporting that the aerial parts of *M. frigidus* are primarily composed of phenolic compounds, particularly flavonoids and terpenoids. Specifically, MFAq exhibited a phenolic content of 84.19 ± 2.10 mg/g (expressed as tannic acid equivalents) and a flavonoid content of 81.66 ± 4.45 mg/g (expressed as rutin equivalents) [2]. In contrast, LCHA showed a phenolic content of 48.93 ± 7.05 µg/mg (in tannic acid equivalents) and a terpene content of 105.90 ± 1.88 µg/mg (in lupeol equivalents) [7]. *L. camara* has been described as containing primarily phenolic compounds and terpenoids [12, 13].

Phenolic compounds are widely recognized as key phytochemicals with antioxidant activity. These compounds exhibit a broad spectrum of applications and contribute to the mitigation of disorders related to oxidative stress [14]. In contrast, terpenoids constitute a structurally diverse class of metabolites with a wide range of reported biological activities, including anti‐inflammatory, antioxidant, antiallergic, and analgesic effects [15]. Therefore, the levels identified in the present study reinforce the biological potential of the extract associations.

The presence of phenolic compounds and terpenes in these extracts suggests a high degree of biological potential, which is consistent with the observed biological activities of the extracts. Consequently, these associations hold considerable promise in the development of a multifunctional formulation for the management of inflammatory skin disorders.

#### 2.1.2 Cell Viability

Macrophages were selected for the cell viability assay due to their critical role in immune responses and their sensitivity to cytotoxic agents, making them appropriate models for initial biocompatibility assessments. As reported by [16], the reduction of MTT to formazan occurs exclusively in metabolically active cells, and the amount of formazan formed is directly proportional to the number of viable cells. According to NBR ISO 10993‐5:2009 (Brazilian Standard—International Organization for Standardization) [17], the cell viability assay should be among the first evaluations to determine the biocompatibility and toxicity of a substance, and in vitro cytotoxicity results must not fall below 70%.

As shown in Figure 1, which presents the results expressed as cell viability percentages, the five MFAq:LCHA associations did not exhibit cytotoxicity when compared with the negative control, with no values below 70% viability in assays using peritoneal macrophages. These findings indicate that the tested associations are biocompatible and safe for potential biological applications, aligning with the established regulatory guidelines for medical devices and materials.

**FIGURE 1:**
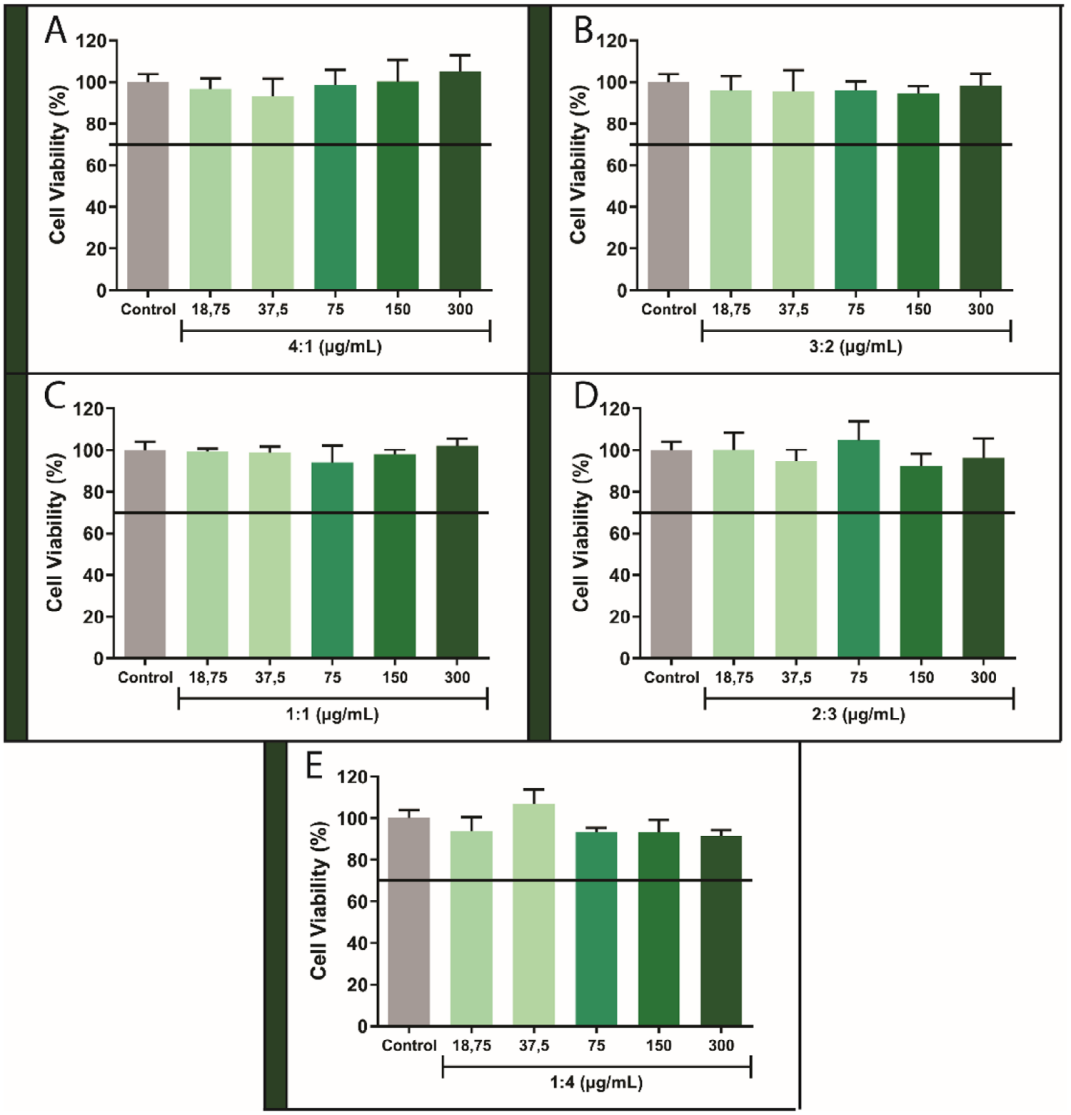
Assessment of the peritoneal macrophages viability after treatment with MFAq:LCHA associations, namely 4:1 (A), 3:2 (B), 1:1 (C), 2:3 (D) and 1:4 (E) at different concentrations (18.75–300 µg/mL). Data are presented as mean ± standard deviation (SD) of two independent experiments performed in triplicate (*n* = 6). Statistical analysis was performed using one‐way ANOVA followed by Bonferroni's post hoc test. Differences were considered statistically significant at *p* < 0.05. The horizontal line indicates the minimum viability threshold of 70%.

According to Mayrink et al. [7], cell viability in peritoneal macrophages remained above 80% at all tested concentrations of LCHA (18.75–300 µg/mL), indicating an absence of cytotoxic effects in this cell lineage. In L929 fibroblasts, viability remained above 70% up to a concentration of 150 µg/mL, demonstrating a concentration‐dependent decrease.

The present study observed no cytotoxicity in the associations, suggesting a potential protective or modulatory effect of the *M. frigidus* constituents. This finding is particularly relevant as it suggests that the combination of extracts could reduce potential adverse effects. Previous studies with MFAq also reported no toxicity in J774A.1 cells using the MTT assay [2], further supporting the biocompatibility of *M. frigidus* and the synergistic effect observed in the associations.

#### 2.1.3 Anti‐Inflammatory Activity

Nitric oxide (NO) is a reactive oxygen species capable of readily diffusing across cell membranes due to its lipophilic nature [18]. It plays a central role in inflammation, as its overproduction is often induced by pro‐inflammatory cytokines, which stimulate immune cells to increase NO synthesis [19]. While NO is a key mediator of the inflammatory response, excessive production during exacerbated inflammation can lead to significant tissue damage [20]. Therefore, quantifying NO provides insight into the anti‐inflammatory potential of bioactive compounds and also reflects antioxidant capacity due to its reactivity.

The formation of lipid droplets (LDs) during inflammation is indicative of a dynamic cellular response, thus highlighting their role not only as lipid storage organelles but also as active platforms for eicosanoid synthesis and inflammatory signaling. The accumulation of LDs in immune cells is recognized as a hallmark of inflammatory diseases, thus rendering LDs potential targets for anti‐inflammatory interventions. The formation of LDs in leukocytes and other cell types occurs during periods of inflammation, and these structures are closely associated with arachidonic acid metabolism. LDs function as sites for the synthesis of key inflammatory mediators, including prostaglandins and leukotrienes [21]. Consequently, the measurement of NO production and LD accumulation provide complementary information, thus enabling a more comprehensive assessment of the anti‐inflammatory potential of natural extracts.

In the present study, NO production (Figure 2A) was differentially modulated by the MFAq:LCHA associations. The 4:1 and 3:2 ratios reduced significantly NO only at the highest concentration (300 µg/mL), 73.62% ± 23.45% and 84.06% ± 30.12%, respectively. In contrast, associations enriched in LCHA, namely 1:1, 2:3, and 1:4, decreased significantly NO levels across multiple concentrations: 300 µg/mL (1:1 = 88.83% ± 19.34%, 2:3 = 87.16% ± 15.55%, 1:4 = 86.48% ± 15.67%), 150 µg/mL (1:1 = 74.84% ± 38.04%, 2:3 = 81.34% ± 32.31%, 1:4 = 72.01% ± 32.32%), and 75 µg/mL (1:1 = 49.97% ± 24.88%, 2:3 = 45.93% ± 28.05%, 1:4 = 62.68% ± 32.68%).

**FIGURE 2:**
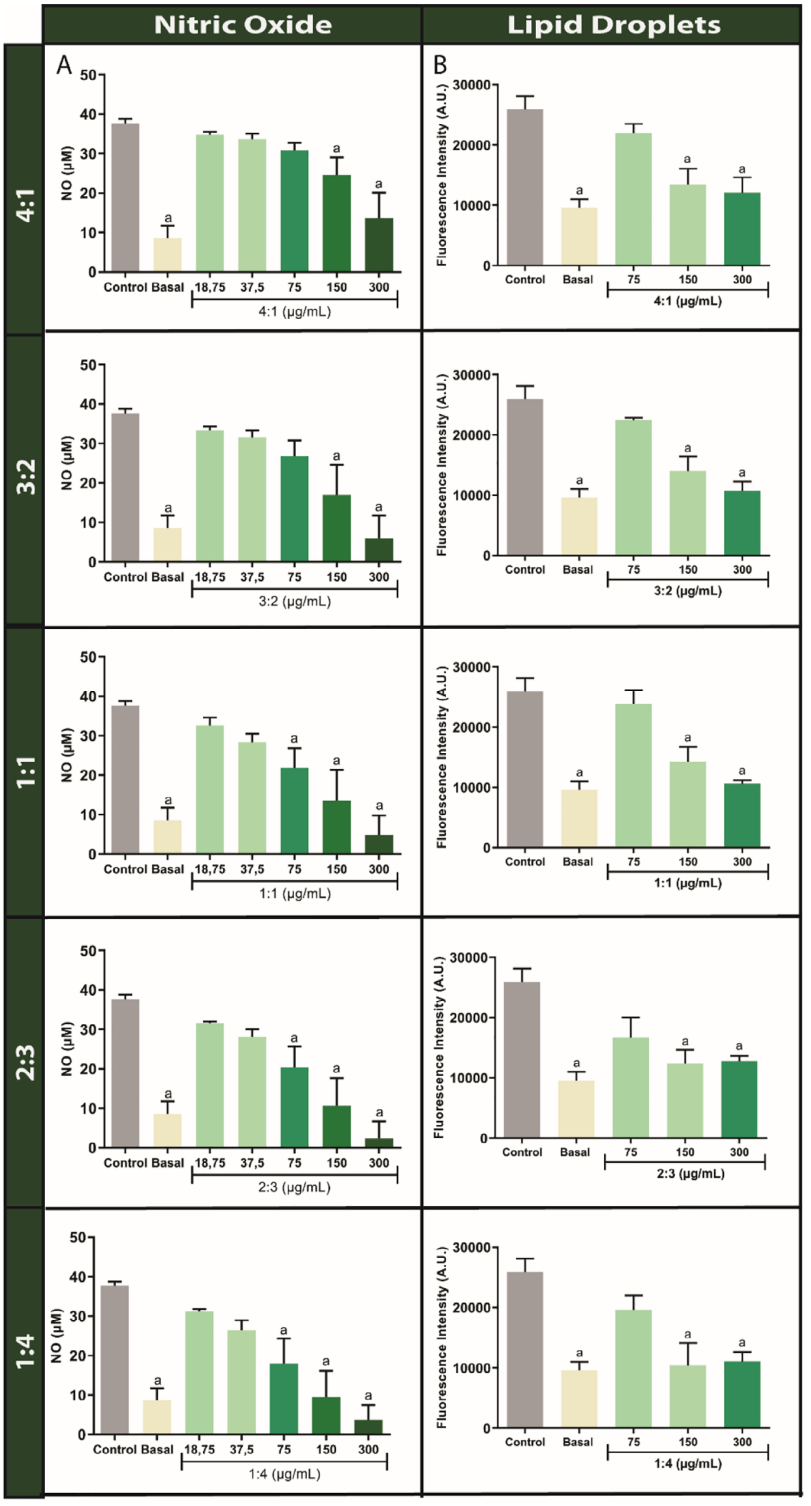
Anti‐inflammatory activity of MFAq:LCHA associations. Nitric oxide (A) and lipid droplets (B) cellular production after treatment at different concentrations (18.75–300 µg/mL). Data are presented as mean ± standard deviation (SD) of two independent experiments performed in triplicate (*n* = 6). Statistical analysis was performed using one‐way ANOVA followed by Bonferroni's post hoc test. Differences were considered statistically significant at *p* < 0.05. Differences were considered statistically significant at *p* < 0.05. a—statistically different from the control group.

Similarly, LD accumulation (Figure 2B) was markedly reduced by all associations, with the most pronounced effects observed at 300 µg/mL (1:1 = 59.48% ± 5.36%, 4:1 = 53.24% ± 16.91%, 3:2 = 58.55% ± 11.70%, 2:3 = 50.53% ± 6.38%, 1:4 = 57.17% ± 10.85%) and 150 µg/mL (1:1 = 44.81% ± 16.35%, 4:1 = 47.90% ± 17.39%, 3:2 = 45.99% ± 16.31%, 2:3 = 52.22% ± 15.47%, 1:4 = 59.92% ± 25.08%) compared to the negative control.

These results are consistent with previous findings for the individual extracts. For instance, Mayrink et al. [7] reported that LCHA has been shown to reduce NO production by up to 100% (300 µg/mL), with significant reductions of 96.09% ± 2.95% and 96.98% ± 4.04% at 75 and 150 µg/mL concentrations, respectively. Concurrently, it reduced LD accumulation by 68.23% ± 3.94% at the lowest concentration (75 µg/mL) and 68.61% ± 3.26% at the highest concentration evaluated (300 µg/mL). Concurrently, earlier research has evidenced the anti‐inflammatory properties of MFAq, which culminated in a NO reduction of approximately 78.13% ± 1.42% at the maximum concentration assessed (200 µg/mL). Moreover, it exhibited in vivo efficacy in ear edema assays [2].

The observed differential modulation of NO production across the associations suggests a concentration‐dependent and composition‐specific anti‐inflammatory effect. The enhanced efficacy of LCHA‐enriched combinations (1:1, 2:3, and 1:4) at lower concentrations suggests stronger inhibitory capacity, potentially resulting from higher LCHA concentration or synergistic interaction among bioactive compounds. The consistent reduction in both NO production and LD accumulation across all associations, particularly at higher concentrations, reinforces the anti‐inflammatory potential of the extract combinations. This parallel inhibition suggests a coordinated action on distinct yet interconnected inflammatory pathways, where the suppression of NO may indirectly impact LD biogenesis, or vice versa, or both are simultaneously targeted by the bioactive compounds.

Overall, these results suggest that extract combinations with higher ratios of LCHA (1:1, 2:3, and 1:4) exhibit the strongest anti‐inflammatory activity, indicating that *L. camara* plays a pivotal role in mediating these effects. These findings are consistent with previous studies reporting promising anti‐inflammatory properties of *L. camara* [7, 22], which are often attributed to its high terpenoid content [13, 23]. In the present study, the levels of terpenoids in the extract combinations ranged from 8.65% to 10.57%, which is likely to have contributed to the reduction in NO production and LD accumulation observed.

These findings underscore the significant therapeutic potential of the *M. frigidus* and *L. camara* association as novel anti‐inflammatory agents. Their ability to simultaneously modulate NO production and LD accumulation suggests a multi‐target approach to inflammation management, which is highly desirable in complex inflammatory conditions.

#### 2.1.4 Antioxidant Activity

Antioxidant molecules are capable of preventing or delaying the oxidation of other compounds, often through different mechanisms [7]. In this study, two complementary assays were employed to evaluate the antioxidant potential of the extracts: DPPH radical scavenging and the β‐carotene/linoleic acid system. The DPPH assay measures the ability of the compound to donate hydrogen atoms to stabilize free radicals, where a higher radical reduction corresponds to lower IC_50_ values and stronger antioxidant activity [7, 24]. The β‐carotene assay evaluates the inhibition of lipid peroxidation, in which antioxidants prevent oxidative degradation of β‐carotene caused by radicals generated from linoleic acid [9].

Previous studies have shown that MFAq exhibited an IC_50_ of 6.19 ± 0.98 µg/mL in the DPPH assay. In the β‐carotene assay, it demonstrated a lipid peroxidation inhibition percentage of 57.29% ± 3.59% and F1 and F2 values of 0.58 ± 0.05 and 1.21 ± 0.24, respectively [2]. In contrast, LCHA showed an IC_50_ of 88.79 ± 17.64 µg/mL in the DPPH assay. For the β‐carotene assay, LCHA exhibited an IC_50_ greater than 38.46 µg/mL (not determined) and a lipid peroxidation inhibition percentage of 41.94% ± 0.11%, with F1 and F2 values of 0.36 ± 0.05 and 1.20 ± 0.04, respectively, where the F2 value was outside the ideal range for good antioxidant activity [7].

In the DPPH assay (Table 2), MFAq‐rich MFAq:LCHA associations (1:1, 3:2, and 4:1), exhibited the lowest IC_50_ values (15.42 ± 1.92, 13.57 ± 0.47, and 11.23 ± 2.16 µg/mL, respectively), indicating superior radical scavenging capacity. Similarly, in the β‐carotene assay, the 1:1 and 3:2 ratios showed IC_50_ values (6.65 ± 0.68 and 7.49 ± 1.01 µg/mL, respectively) that did not differ significantly from rutin (*p <* 0.05). Moreover, the inhibition percentages of the 4:1, 3:2, and 2:3 associations (61.99% ± 3.11%, 56.98% ± 3.78%, and 55.86% ± 1.45%, respectively) were comparable to the control (61.67% ± 1.33%) (*p <* 0.05).

**TABLE 2:** Antioxidant activity of MFAq:LCHA associations, evaluated by DPPH and β‐carotene assay.

	DPPH	β‐Carotene assay			
Associations	IC_50_ (µg/mL)	IC_50_ (µg/mL)	% Inhibition (38.46 µg/mL)	F1	F2
4:1	11.23 ± 2.16^d^, ^e^, ^f^	8.56 ± 0.94^e^, ^f^	61.99 ± 3.11^c^, ^f^	0.24 ± 0.02^e^, ^f^	0.38 ± 0.08
3:2	13.57 ± 0.47^d^, ^e^, ^f^	7.49 ± 1.01^e^	56.98 ± 3.78 ^e^	0.31 ± 0.01	0.47 ± 0.01
1:1	15.42 ± 1.92^d^, ^e^, ^f^	6.65 ± 0.68^e^	52.47 ± 0.19^a^, ^f^	0.31 ± 0.02	0.51 ± 0.02
2:3	22.12 ± 3.03^a^, ^b^, ^c^, ^f^	8.7 ± 1.92^e^, ^f^	55.86 ± 1.45	0.30 ± 0.01	0.48 ± 0.07
1:4	39.15 ± 1.92^a^, ^b^, ^c^, ^f^	> 38.46^a^, ^b^, ^c^, ^d^, ^f^	47.12 ± 1.71^a^, ^b^, ^f^	0.35 ± 0.02^a^	0.51 ± 0.06
Rutin	0.44 ± 0.22	6.27 ± 0.31	61.67 ± 1.33	0.34 ± 0.02	0.58 ± 0.12

*Note*: Data are presented as mean ± standard deviation (SD) of three independent experiments performed in triplicate (*n* = 9). Statistical analysis was performed using one‐way ANOVA followed by Bonferroni's post hoc test. Differences were considered statistically significant at *p* < 0.05. Different letters indicate statistically significant differences between groups:

^a^ different from 4:1;

^b^ different from 3:2;

^c^ different from 1:1;

^d^ different from 2:3;

^e^ different from 1:4;

^f^ different from Rutin.

Further evaluation using F1 and F2 parameters, reflecting antioxidant effectiveness in the initiation and propagation phases of lipid oxidation, revealed that all combinations fell within the expected range for strong activity (0–1) and were statistically similar to the rutin, except for the F1 value of 4:1 (0.24 ± 0.02). MFAq:LCHA associations 1:1, 3:2, and 4:1 consistently displayed the lowest F1 and F2 values, highlighting their ability to block both early and propagative oxidative reactions more effectively than the standard.

The consistent display of low F1 and F2 values across the most active combinations is particularly significant, as it indicates a dual‐action antioxidant mechanism. A low F1 value reflects a potent ability to scavenge initial free radicals and prevent the onset of lipid peroxidation, while a low F2 value signifies effective quenching of propagating radicals, thereby halting the chain reaction of oxidative damage [7, 25]. This comprehensive protection against both the early and late stages of lipid oxidation highlights the robust antioxidant profile of these associations.

Overall, extract combinations with higher MFAq content (1:1, 3:2, and 4:1) demonstrated the most pronounced antioxidant effects. These results are consistent with literature reports, indicating that *M. frigidus* is rich in phenolic compounds, particularly flavonoids, and terpenes, which are known for their antioxidant properties [26]. Consequently, the observed antioxidant potential of the combinations can be directly attributed to the high MFAq content, supporting its role as the main contributor to the activity.

The strong correlation between the DPPH radical scavenging activity and the inhibition of lipid peroxidation in the β‐carotene/linoleic acid system further validates the comprehensive antioxidant potential of the MFAq‐rich combinations. This consistency across different mechanisms of action (radical scavenging vs. lipid peroxidation inhibition) suggests a broad‐spectrum antioxidant capacity, likely due to the diverse array of phytochemicals present.

These findings highlight the significant potential of *M. frigidus* and *L. camara* associations as natural sources of antioxidants for various applications, including functional foods, nutraceuticals, and pharmaceutical formulations aimed at mitigating oxidative stress‐related diseases.

#### 2.1.5 Selection of the Most Effective Association and Its Phytochemical Profile

From the assays performed with the five different MFAq:LCHA associations, and based on the previously discussed anti‐inflammatory analyses, the 1:1, 2:3, and 1:4 ratios stood out, exhibiting superior and consistent biological responses. This enhanced anti‐inflammatory effect appears to be related to the higher relative content of LCHA in these combinations, which is rich in terpenes, bioactive molecules widely recognized for their anti‐inflammatory activity [13, 27]. Conversely, the antioxidant assays highlighted the 4:1, 3:2, and 1:1 ratios as the most active, likely due to the greater contribution of MFAq. This extract contains abundant phenolic compounds, flavonoids, and terpenes, which are well‐known for their strong antioxidant capacity [26].

Among all combinations, the 1:1 association consistently exhibited the best overall performance. It achieved reductions of 88.83% ± 19.34% and 59.48% ± 5.36% in NO production and LD levels, respectively, values superior to other promising ratios (2:3 and 1:4), demonstrating its marked anti‐inflammatory potential. Concurrently, it reached IC_50_ values of 15.42 ± 1.92 and 6.65 ± 0.68 in the DPPH and β‐carotene antioxidant assays, comparable to those observed for the best antioxidant‐performing ratios (3:2 and 4:1). These results provide a strong empirical basis for its selection.

The choice of the 1:1 ratio, providing equal proportions of MFAq and LCHA, offers distinct scientific and practical advantages. Beyond its balanced biological profile, this ratio facilitates standardization, enhances reproducibility, and may improve cost‐effectiveness for future large‐scale applications. Moreover, it suggests an optimal synergistic interaction between the bioactive constituents of both extracts, maximizing the therapeutic potential through the complementary mechanisms of their compounds.

Therefore, based on the comprehensive biological assessment, the MFAq:LCHA 1:1 association was selected as the most promising candidate for further studies and for incorporation into the bioadhesive film formulations. To elucidate the molecular basis underlying its potent anti‐inflammatory and antioxidant activities, a phytochemical profiling of the selected ratio was conducted. This analysis aimed to correlate the identified compounds with the observed biological effects and to provide mechanistic insights into the synergistic contributions of each extract.

The chromatographic profile obtained by UFLC‐QTOF‐MS is shown in Figure S1. A total of 12 compounds were identified in the MFAq:LCHA (1:1) association, comprising six terpenes, five flavonoids, and one anthraquinone (Table 3). The terpenes identified—ursolic acid, lamiide, harounoside, lantadene C, geniposide, and clarinoside—have been extensively reported for their anti‐inflammatory and wound‐healing activities [7, 28]. The flavonoids identified, pectolinarin, quercetin‐hexosyl‐pentoside, rutin, quercetin‐hexoside, and kaempferol‐3‐O‐rutinoside, are also well‐documented for their antioxidant and skin‐protective properties [29]. Additionally, the identified anthraquinone (2‐azaanthraquinone) has been associated with both anti‐inflammatory and wound‐healing effects [30, 31].

**TABLE 3:** Compounds identified in the MFAq:LCHA 1:1 association by UFLC‐QTOF‐MS.

N^a^	Rt (min)^a^	Fragment of molecular ions MS_2_ (m/z)	Compound name and molecular structure	Originating extract	References
1	2.698	458.1926 (M + 2H)	Ursolic acid	LCHA	[32]
2	10.584	867.2815 (M + Na); 423.1554 (M + H)	Lamiide	LCHA	[32]
3	10.981	539.1832 (M + H); 377.1293 (M + H‐Hexose); 215.0735 (M + H‐hexose‐hexose)	Harounoside	MFAq	[33]
4	12.555	556.2109 (M + 2H)	Lantadene C	LCHA	[32]
5	13.466	406.2128 (M + NH_4_)	Geniposide	LCHA	[32]
6	13.913	623.1316 (M + H)	Pectolinarin	LCHA	[32]
7	14.029	540.2145 (M + NH^3^); 361.1338 (M + H‐hexose); 215.0731 (M + H‐hexose‐ramnose)	Clarinoside	MFAq	[33]
8	14.725	597.1514 (M + H); 465.1073 (M + H‐pentose); 303.0541 (M + H‐pentose‐hexose)	Quercetin‐hexosyl‐pentoside	MFAq	[33]
9	14.907	611.1690 (M + H); 465.1082 (M + H‐ramnose); 303.0541 (M + H‐ramnose‐glucose)	Rutin	MFAq	[33]
10	15.305	465.1083 (M + H); 303.0543 (M + H‐hexose)	Quercetin‐hexoside	MFAq	[34]
11	15.620	595.1730 (M + H); 449.1136 (M + H‐ramnose); 287.0590 (M + H‐ramnose‐glucose)	Kaempferol‐3‐O‐rutinoside	MFAq	[33]
12	21.981	210.0555 (M + H)	2‐Azaanthraquinone	MFAq	[33]

^a^ Retention time (Rt) in minutes.

Analyzing the most probable origin of each compound, it was inferred that ursolic acid, lamiide, lantadene C, and geniposide derive predominantly from LCHA, while harounoside and clarinoside originate from MFAq. Among the flavonoids, pectolinarin is primarily attributed to LCHA, whereas the remaining ones are mainly found in MFAq. The 2‐azaanthraquinone appears to be exclusive to MFAq.

The co‐occurrence of bioactive compounds from both extracts provides a robust molecular explanation for the observed synergistic effects. Specifically, the combination of anti‐inflammatory terpenes (mostly from LCHA) with antioxidant flavonoids (largely from MFAq) suggests a multitargeted mechanism, where distinct yet complementary pathways are modulated to counteract inflammation and oxidative stress simultaneously.

In summary, the comprehensive phytochemical profile of the selected MFAq:LCHA (1:1) association, enriched with compounds known for their anti‐inflammatory, antioxidant, and wound‐healing properties, offers a solid scientific ground for its inclusion in bioadhesive film formulations. This strategic formulation aims to harness the synergistic potential of both extracts, providing enhanced therapeutic efficacy for topical applications, particularly in the management of wound healing and inflammatory skin conditions.

### 2.2 Characterization of Film Precursor Solutions

#### 2.2.1 Electrical Conductivity, Zeta Potential, and Determination of Particle Size Distribution Using Dynamic Light Scattering (DLS)

The analysis of precursor solutions prior to film casting provided important insights into their ionic behavior, colloidal stability, and nanoscale organization [3]. Electrical conductivity (EC), zeta potential (ZP), and hydrodynamic diameter (*D*_h_) are interrelated parameters that collectively describe the electrostatic environment and particle dispersion within polymeric systems [35, 36].

The EC results (Figure 3A) for the Film‐BL solution, composed of chitosan, acetic acid, and glycerol, confirmed its electrolytic character, mainly attributed to the protonation of chitosan amino groups under acidic conditions [37]. The incorporation of MFAq:LCHA associations at concentrations between 0.5%, 1.0%, and 2.5% significantly increased EC values compared to the blank (*p <* 0.05), with respective increases of 49.68% ± 1.53%, 89.14% ± 0.89%, and 75.85% ± 1.10%. This increase is likely associated with the presence of ionizable constituents in the extracts, such as phenolic acids and organic ions, which enhance the ionic strength and charge mobility in the system [38]. These findings are in line with previous reports where the addition of plant extracts to chitosan‐based solutions promoted an increase in electrical conductivity due to the presence of soluble ionic species [3, 39]. However, a slight reduction in conductivity observed at 2.5% extract concentration suggests that excessive ionic content may promote ion‐ion interactions or partial complexation, reducing ion mobility and overall conductivity.

**FIGURE 3:**
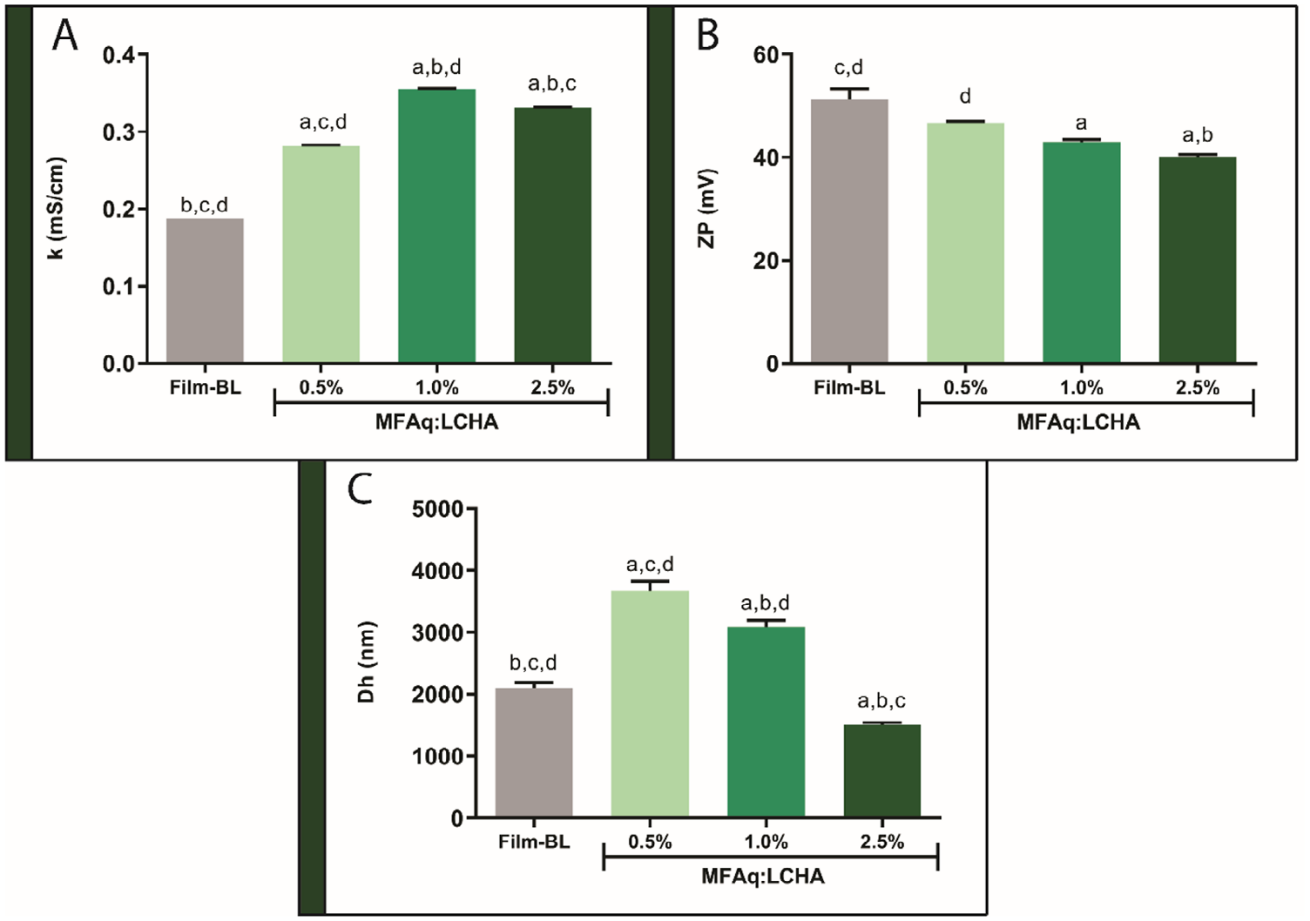
Electrical conductivity (A), zeta potential (B), and hydrodynamic diameter (C) values of the precursor solutions of chitosan films containing the MFAq:LCHA associations (MFAq:LCHA‐0.5%–2.5%) and blank film (Film‐BL). Data in panels A and B are presented as mean ± standard deviation (SD) of five independent measurements for each sample (*n* = 5). Data in panel C are presented as mean ± SD of ten independent measurements, with ten runs performed for each measurement (*n* = 10). Statistical analysis was performed using one‐way ANOVA followed by Bonferroni's post hoc test. Differences were considered statistically significant at *p* < 0.05. Different letters indicate statistically significant differences between groups: a—different from Film‐BL; b—different from MFAq:LCHA‐0.5%; c—different from MFAq:LCHA‐1.0%; d—different from MFAq:LCHA‐2.5%.

Regarding the ZP values (Figure 3B), all precursor solutions exhibited positive charges above +40 mV, indicative of excellent colloidal stability [3]. The blank solution (Film‐BL) presented high positive potential values consistent with the cationic nature of chitosan, whose amino groups remain protonated in acidic medium [36, 40]. The progressive reduction in ZP values upon extract association incorporation (*p <* 0.05) can be attributed to the partial neutralization of chitosan's amino groups by negatively charged components from the extracts. Such behavior evidenced electrostatic interactions between the polymer chains and bioactive molecules, as previously described for chitosan‐phenolic systems [39]. Considering that MFAq:LCHA associations contain abundant phenolic compounds and terpenes, a plausible mechanism involves charge neutralization or hydrogen bonding between phenolic hydroxyl groups and protonated amines of chitosan [41]. This interaction modulates the polymer's surface charge, supporting the observed ZP decrease without compromising the overall stability of the system.

The *D*_h_ results (Figure 3C) revealed a concentration‐dependent behavior. The addition of 0.5% association led to a significant increase in particle size compared to the blank (*p <* 0.05), indicating initial aggregation or loose association among chitosan chains and extract molecules. However, at higher extract concentrations (1%–2.5%), *D*_h_ values decreased, suggesting a more homogeneous and dispersed system. This reduction may be associated with the increase in ionic strength and partial neutralization of chitosan charges, which reduce interchain interactions and aggregation [36].

Integrating the D_h_ and electrical conductivity results, it can be inferred that higher extract concentrations in the precursor solutions lead to increased electrical conductivity and, consequently, to smaller dispersed particle sizes and a reduced likelihood of aggregate formation. The presence of aggregates (larger molecular assemblies) would otherwise contribute to increased viscosity within the system. Altogether, these results demonstrate that the incorporation of MFAq:LCHA extracts modulates the electrostatic balance and molecular organization of the chitosan‐based precursor solutions. The combined analysis of EC, ZP, and D_h_ suggests that electrostatic interactions between chitosan and phytochemical components govern the stability, ionic mobility, and particle size distribution of the system, ultimately influencing the microstructural uniformity and stability of the resulting films.

#### 2.2.2 Rheological Parameters

Rheology is the science that studies the flow and deformation of matter. Understanding rheological parameters is therefore essential for the development of formulations, as it enables the investigation of possible intermolecular interactions and their influence on flow properties, viscosity, deformation, and sample stability [3, 42]. Accordingly, the rheological characteristics of the precursor solutions were investigated to predict their film‐forming capacity and related properties [3, 43].

Based on the viscoelastic profiles shown in Figure 4A,B, all precursor solutions exhibited a liquid‐like viscoelastic behavior, characterized by a viscous modulus (G'') greater than the elastic modulus (*G*') and tan *δ* values exceeding 1. Among the formulations, MFAq:LCHA‐2.5% exhibited the most pronounced liquid‐like behavior, suggesting that increasing extract concentration disrupts the organization of the chitosan polymeric network, thereby reducing its structural integrity.

**FIGURE 4:**
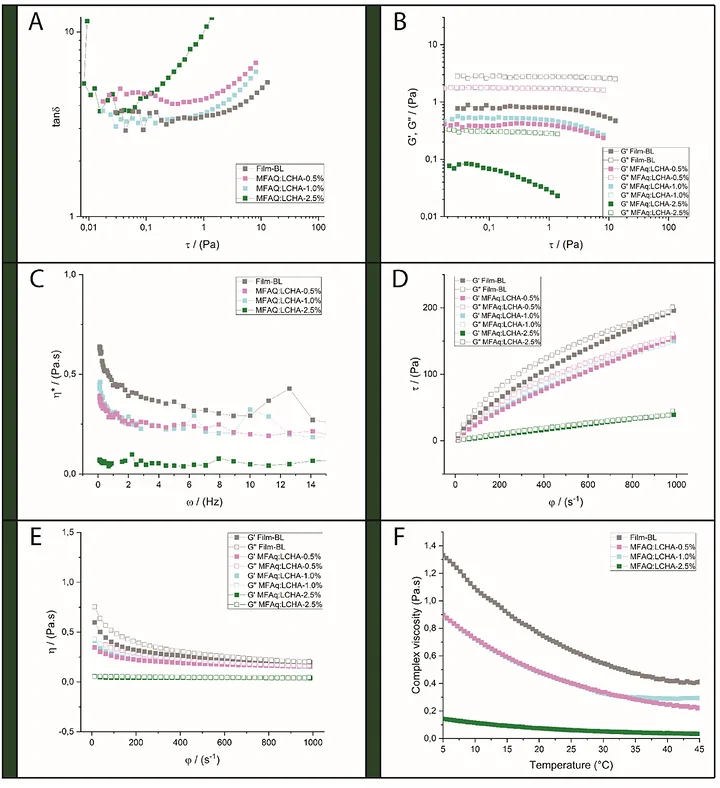
Rheological analysis of chitosan films containing the MFAq:LCHA associations (MFAq:LCHA‐0.5%–2.5%) and blank film (Film‐BL). Phase angle tangent (*δ*) (A). Storage (*G*′) and loss (*G*″) moduli (B). Complex viscosity versus frequency (C). Steady‐state flow (D) and viscosity (E) curves. Viscosity versus temperature (5°C–45°C) (F). Curves were processed using OriginPro 9.0 software.

The assessment of complex viscosity, derived from the material's response to oscillatory deformation, offers valuable insights into its flow and deformation behavior under stress. As illustrated in Figure 4C, the η* values decreased with increasing shear frequency, indicating a pseudoplastic (shear‐thinning) profile. This trend suggests a progressive disruption of intermolecular interactions within the system under shear. Notably, MFAq:LCHA‐2.5% exhibited a lower initial viscosity compared with other concentrations, with only a slight reduction as shear frequency increased. This response may be attributed to the higher extract concentration, which likely disturbed the chitosan polymeric matrix, enhancing molecular mobility and consequently increasing the formulation's fluidity.

These data are in accordance with the findings of Silva et al. [39], who evaluated the rheological properties of chitosan polymer films containing hydroalcoholic extract of *Coffea arabica* and found that the addition of extract components leads to a reduction in viscosity, attributing this to the gradual formation of discrete aggregates, where the smaller particle sizes lead to a reduction in friction between the chitosan chains, leading to lower viscosity [39]. Conversely, lower extract concentrations (MFAq:LCHA‐0.5% and MFAq:LCHA‐1%) promoted synergistic interactions with chitosan without altering its pseudoplastic behavior.

Flow and viscosity curves as a function of shear rate provide valuable insights into the material's response to increasing and subsequently removing applied stress. As shown in Figure 4D,E, the formulations exhibited thixotropic behavior, characterized by a reduction in viscosity and enhanced flow under deformation. Upon cessation of shear, the system progressively recovered its structure, and viscosity returned to near‐initial values. The incorporation of MFAq:LCHA reduced the degree of thixotropy, as evidenced by the progressive decrease in the area between the ascending and descending curves with increasing extract concentration. Moreover, the ascending curve of MFAq:LCHA‐2.5% consistently displayed lower viscosity across the entire shear range compared with other precursor solutions, reinforcing that higher extract concentrations promote increased formulation fluidity. Overall, all formulations exhibited rheological behavior typical of non‐Newtonian thixotropic materials [44].

These findings are consistent with literature reports, which suggest that the addition of plant extracts may disrupt the three‐dimensional intermolecular interactions among chitosan macromolecules. This disruption leads to the formation of smaller and more discrete particles, thereby decreasing viscosity and pseudoplasticity [3, 39]. Such structural modifications explain the observed enhancement in flow and reduced resistance to deformation under shear.

An increase in temperature enhances molecular mobility and weakens intermolecular interactions, leading to reduced viscosity and increased fluidity of the samples [45]. Accordingly, the evaluation of viscosity as a function of temperature (Figure 4F) provided insights into the thermal behavior of the precursor solutions. Within the 20°C–40°C range, representative of typical Brazilian environmental conditions, no abrupt changes in viscosity were detected (Film‐BL: 0.34 Pa·s; MFAq:LCHA‐0.5%: 0.23 Pa·s; MFAq:LCHA‐1.0%: 0.18 Pa·s; MFAq:LCHA‐2.5%: 0.03 Pa·s). These results indicate that the formulations maintain rheological stability under ambient temperatures, which is a desirable feature for practical applications involving storage, handling, and transport [43]. Similar findings were reported by Fajardo et al. [3], whose chitosan‐based polymeric films also exhibited consistent thermal stability at room temperature.

### 2.3 Characterization of Films Containing MFAq:LCHA

#### 2.3.1 In Vitro Drug Release Experimentation

The release study was conducted to evaluate the efficiency of phytoconstituent release from the MFAq:LCHA‐containing polymeric films. A system was designed to mimic the contact of the films with the skin and their unidirectional release. This assay is of great importance, since the release of the active compounds is essential for them to interact with the skin and exert their pharmacological effect.

The obtained profile (Figure 5) shows that MFAq:LCHA‐0.5% reached 100% release after 40 min of the experiment, whereas MFAq:LCHA‐1% achieved complete release within 180 min, and MFAq:LCHA‐2.5% within 90 min. These results can be attributed to the high solubility of the isolated extracts (MFAq and LCHA) and their MFAq:LCHA associations in aqueous media, which was evident during the preparation of the sink condition for the release assay. In this case, the sink condition refers to the minimum amount of medium required to solubilize MFAq:LCHA before reaching saturation, with a minimum solubility value of 7.5 mg/mL.

**FIGURE 5:**
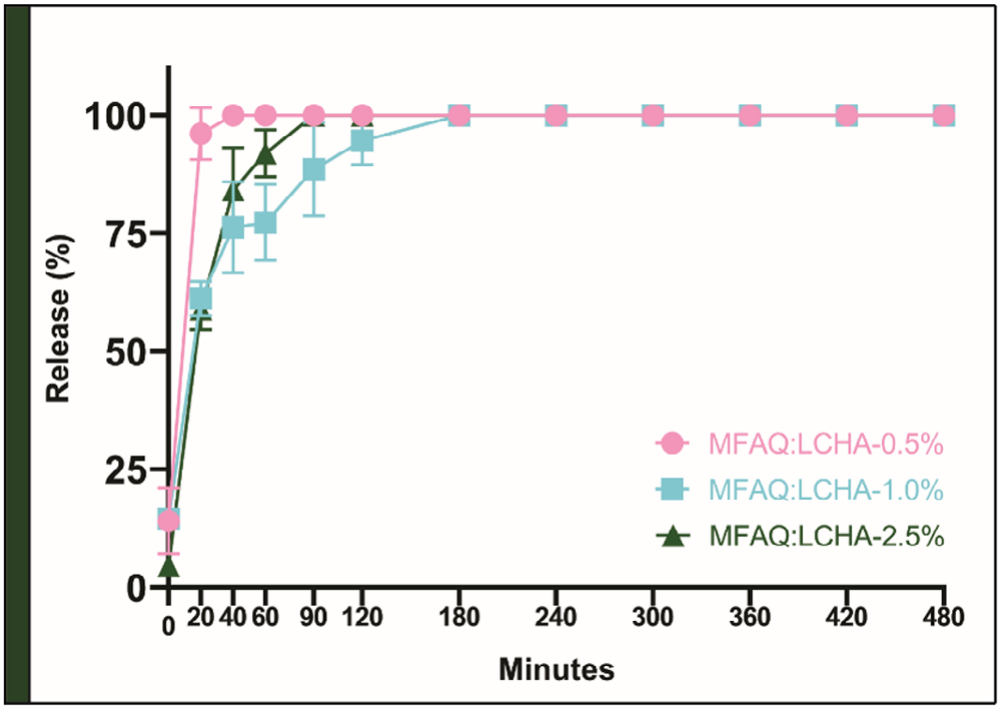
Percentage of extracts released from chitosan films containing the MFAq:LCHA associations (MFAq:LCHA‐0.5%–2.5%) between 0 and 480 min, considering *λ* = 270 nm. Results are expressed as mean ± standard deviation (SD) of triplicate measurements (*n* = 3). The percentage of release was calculated based on the total extract content present in the film fragment used in the assay.

This rapid release of the compounds may be associated with the presence of hydrophilic constituents in MFAq:LCHA, which have an affinity for aqueous environments, being quickly released and dispersed. As previously discussed, MFAq:LCHA contains predominantly phenolic compounds, which are generally hydrophilic due to the presence of free hydroxyl groups, thus exhibiting high aqueous affinity and promoting rapid formulation release. This hypothesis is further corroborated by Silva et al. [39], who demonstrated that enhanced water uptake (evaluated under an aqueous environment) induces structural relaxation of the films, thereby yielding results that align with the present findings, particularly the increased solubilization and diffusion of solutes.

The longer time required to achieve 100% release between the 0.5% and 1% films may be related to the higher extract concentration in the formulation, which requires more time for complete release. Conversely, the reduced release time observed in the 2.5% films may be explained by the structural changes identified in the rheological analyses, where the excess of MFAq:LCHA led to the disorganization of the polymeric matrix, thereby facilitating the release of the phytoconstituents from the formulation.

#### 2.3.2 Morphology

Scanning electron microscopy (SEM) is an advanced approach for characterizing chitosan‐based films, enabling detailed visualization of the surface morphology and internal structure, as well as the distribution of components within the polymeric matrix. In this study, SEM was employed to examine both the surface and cross‐sectional areas of the films to assess the uniformity of extract and chitosan particle distribution and to identify possible morphological changes arising from interactions between different extract concentrations and the polymer. Surface analyses were performed at magnifications of 500× and 3500×, while cross‐sectional analyses were conducted at 400× to 800×.

As shown in Figure 6, the microstructural features of the films at different magnifications reveal distinct morphological patterns. The surfaces of Film‐BL, MFAq:LCHA‐0.5%, and MFAq:LCHA‐1.0% appeared smooth and homogeneous, with no visible aggregates, suggesting efficient solubilization and uniform dispersion of MFAq:LCHA within the chitosan matrix. Cross‐sectional analyses confirmed this trend, as these formulations displayed compact and continuous structures with uniform edges, indicating a well‐organized polymeric network. These morphological characteristics are consistent with the results obtained from electrical conductivity, zeta potential, and hydrodynamic diameter (Dh) analyses.

**FIGURE 6:**
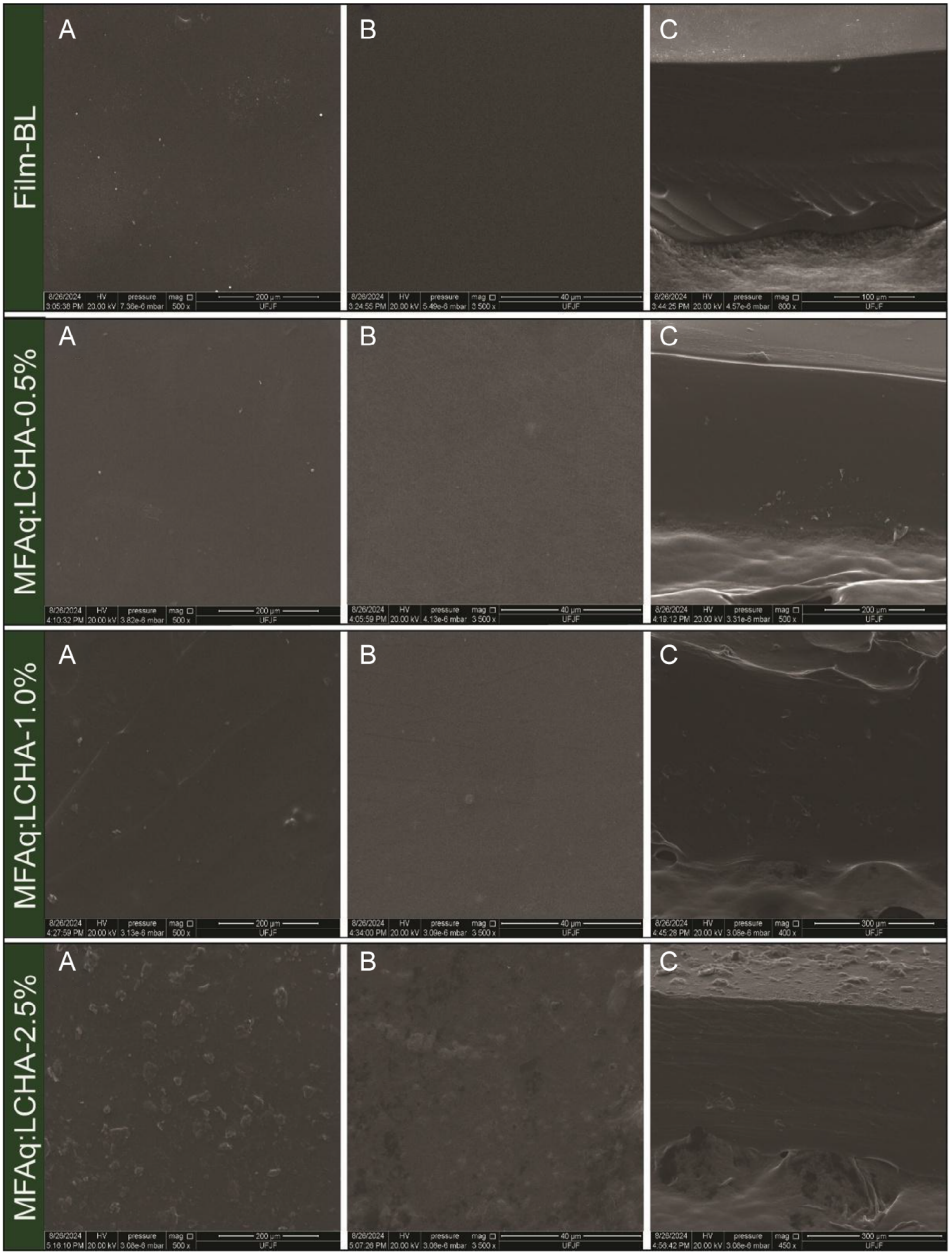
Scanning Electron Microscopy (SEM) from chitosan films containing the MFAq:LCHA associations (MFAq:LCHA‐0.5%–2.5%) and blank film (Film‐BL). Surface micrographs were obtained at magnifications of 500× (A) and 3500× (B), while edge images (C) were acquired at magnifications ranging from 400× to 800×.

In contrast, MFAq:LCHA‐2.5% exhibited distinct aggregates of comparable size and regular distribution, likely due to the excessive extract concentration and subsequent disruption of the polymeric network, as previously discussed in the rheological evaluation. Additionally, the presence of circular, dark, and evenly spaced regions observed in the MFAq:LCHA‐2.5% images suggests the formation of micropores. Such microporous structures may enhance gas exchange and oxygen permeability, potentially providing functional advantages for topical applications.

The present findings are consistent with those reported in the literature, in which 1% chitosan films and extract‐free controls exhibited homogeneous and smooth surfaces. A similar pattern was observed for MFAq:LCHA‐0.5‐1.0% and their respective blanks. Moreover, there are reports that films at higher concentrations displayed irregular surfaces with aggregates, suggesting incomplete incorporation of the extract into the polymeric matrix, which is consistent with the observations for MFAq:LCHA‐2.5% [3].

#### 2.3.3 Weight, Thickness, and Moisture Content

The determination of average weight and thickness is essential for evaluating the uniformity parameters of the films, which, in turn, provides insight into the homogeneous distribution of constituents throughout the film [46]. All films incorporated with extract exhibited significantly higher average weight and thickness values (Table 4) compared to the blank film (average weight: 25.30 ± 2.30 mg; thickness: 0.20 ± 0.03 mm) (*p <* 0.05). These results indicate that the extract concentration in the films directly influences these parameters, with the film containing the highest extract concentration (2.5%) displaying the highest average weight (63.30 ± 4.79 mg) and thickness (0.31 ± 0.02 mm). Based on these findings, it can be hypothesized that the increase in film thickness upon incorporation of the extract association may result from possible aggregation of these compounds with the polymeric matrix, leading to thicker films. Furthermore, it is known that the ideal thickness for skin is comparable to that of the epidermis, ranging from 0.05 to 2 mm; therefore, the developed films may be suitable for such applications [47].

**TABLE 4:** Weight, thickness, and moisture content from chitosan films containing the MFAq:LCHA associations (MFAq:LCHA‐0.5%–2.5%) and blank film (Film‐BL).

	Film‐BL	MFAq:LCHA‐0.5%	MFAq:LCHA‐1.0%	MFAq:LCHA‐2.5%
	Mean ± SD	Mean ± SD	Mean ± SD	Mean ± SD
Weight (mg)	25.30 ± 2.30^b^, ^c^, ^d^	53.20 ± 4.32^a^, ^d^	48.40 ± 4.69^a^, ^d^	63.3 ± 4.79^a^
Thickness (mm)	0.20 ± 0.03^b^, ^c^, ^d^	0.33 ± 0.02^a^, ^c^	0.26 ± 0.01^a^, ^b^, ^d^	0.31 ± 0.02^a^, ^c^
Moisture content (%)	32.32 ± 2.16^b^, ^c^, ^d^	56.93 ± 3.44^a^, ^c^	63.20 ± 1.55^a^, ^b^, ^d^	53.30 ± 0.40^a^, ^c^

*Note*: Data are presented as mean ± standard deviation (SD). For average weight and thickness analyses, ten 1 × 1 cm squares of each film were evaluated, and all measurements were performed in quadruplicate. Moisture content analysis was performed using four 1 × 1 cm film sections in quadruplicate. Statistical analysis was performed using one‐way ANOVA followed by Bonferroni's post hoc test. Differences were considered statistically significant at *p* < 0.05. Different letters indicate statistically significant differences between groups:

^a^ different from Film‐BL;

^b^ different from MFAq:LCHA‐0.5%;

^c^ different from MFAq:LCHA‐1.0%;

^d^ different from MFAq:LCHA‐2.5%.

Film moisture content is an important parameter, as adequate skin hydration is essential to prevent dryness, enhance film adhesion at the application site, and facilitate cellular migration [48, 49]. As shown in Table 4, all films (MFAq:LCHA‐0.5%: 56.93 ± 3.44; MFAq:LCHA‐1.0%: 63.20 ± 1.55; MFAq:LCHA‐2.5%: 53.30 ± 0.40) exhibited significantly higher moisture content than Film‐BL (32.32 ± 2.16) (*p <* 0.05), indicating that the addition of extract enhances the film's moisture retention, potentially improving its applicability and ability to maintain hydration at the application site. These findings can be attributed to the presence of hydrophilic compounds in MFAq:LCHA, as previously discussed, particularly its phenolic content.

#### 2.3.4 Swelling Ratio and Solubility Degree

The liquid absorption capacity is highly relevant for the development of wound‐healing films; this property is crucial for absorbing excess exudates, thereby reducing the risk of infection. Additionally, this functionality is important for maintaining film adhesion at the application site and can influence the release of active compounds into the medium [50]. As shown in Figure 7A, the highest liquid absorption occurred within the first hour of the experiment, followed by stabilization. At the end of the 8‐h experiment, only the MFAq:LCHA‐2.5% film remained intact, without signs of rupture. The Film‐BL film began to show signs of rupture after 6 h, whereas MFAq:LCHA‐0.5% and MFAq:LCHA‐1.0% films started to rupture shortly after 2 h, which complicated their collection. The blank film exhibited the lowest swelling percentage (final value: 37.09 ± 0.01), followed by the 2.5% film (62.61 ± 0.01), 1% (82.27 ± 0.01), and 0.5% (97.44 ± 0.01).

**FIGURE 7:**
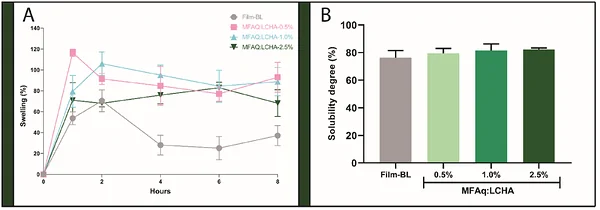
Swelling ratio (A) and solubility degree (B) from chitosan films containing the MFAq:LCHA associations (MFAq:LCHA‐0.5‐2.5%) and blank film (Film‐BL). Data are presented as mean ± standard deviation (SD). Ten 1 × 1 cm film fragments were evaluated for each formulation, and all measurements were performed in quadruplicate. Statistical analysis was performed using one‐way ANOVA followed by Bonferroni's post hoc test. Differences were considered statistically significant at *p* < 0.05. Different letters indicate statistically significant differences between groups: a—different from Film‐BL; b—different from MFAq:LCHA‐0.5%; c—different from MFAq:LCHA‐1.0%; d—different from MFAq:LCHA‐2.5%.

The difficulty in recovering the films, particularly the 0.5% formulation after several hours, may have contributed to the lower observed swelling values for this sample. Furthermore, the swelling capacity of the films is directly related to the amino groups of chitosan, which have a high affinity for forming hydrogen bonds with water [46]. Therefore, the increased swelling capacity observed with the addition of extract can be attributed to the presence of hydrophilic compounds in the extracts, which enhance the hydrophilicity of chitosan. This finding is consistent with previous discussions in other sections, such as the release study and moisture content evaluation.

Another critical parameter is solubility, which plays a key role in understanding the interactions among film components when in contact with exudates or other aqueous fluids [51]. As shown in Figure 7B, the solubility of the polymeric films ranged from 76% to 82%, with no statistically significant differences among the films (*p <* 0.05). This indicates the films’ affinity for aqueous media, further supporting the swelling assay results and corroborating findings from the release study and moisture content evaluation.

#### 2.3.5 Thermogravimetric Analysis

Thermogravimetric analysis (TGA) and derivative thermogravimetry (DTG) are valuable techniques for assessing the thermal stability of chitosan films by monitoring transformations induced by heat, such as dehydration, decomposition, and melting.

As shown in Figure 8A, the DTG curve of pure chitosan exhibits two main degradation events at approximately 180°C and 340°C, which correspond to the depolymerization and decomposition of the chitosan backbone. The first peak is associated with the degradation of the pyranose rings, while the second is attributed to cleavage of glycosidic bonds between glucosamine and N‐acetylglucosamine units [52]. These events coincide with the major mass losses observed in the TGA curve (Figure 8B), occurring between 140°C and 200°C (> 10%) and 280°C–370°C (> 36%).

**FIGURE 8:**
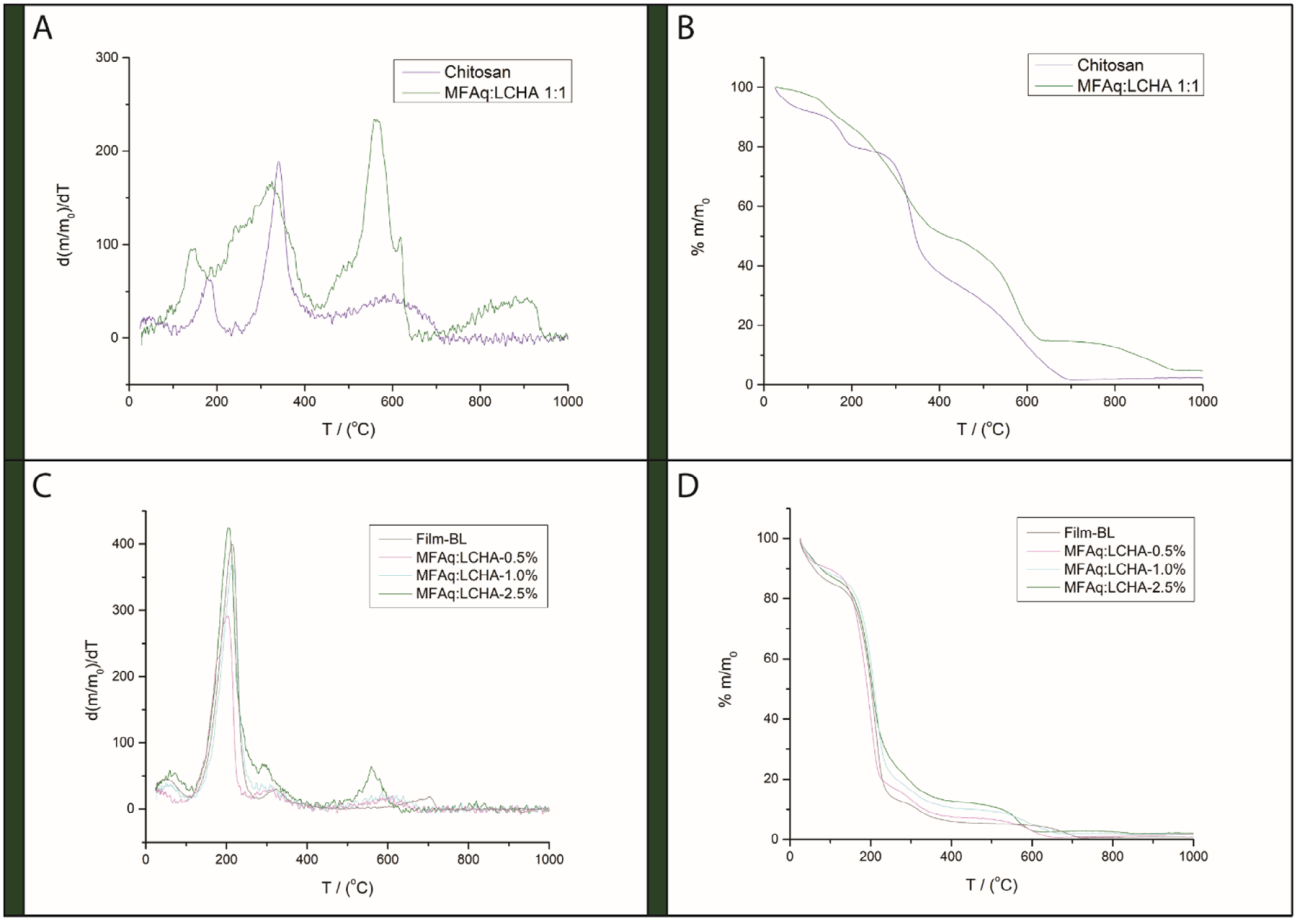
Derivative thermogravimetry (DTG) (A, C) and thermogravimetric analysis (TGA) (B, D) of powdered chitosan and MFAq:LCHA (A, B), and chitosan films containing MFAq:LCHA extract associations (0.5%–2.5%) and blank film (Film‐BL) (C, D), recorded over the temperature range of 0°C–1000°C. Thermal analyses were performed in triplicate (*n* = 3). Curves were processed using OriginPro 9.0 software.

For the MFAq:LCHA extract (Figure 8A), the DTG curve shows an initial minor mass loss (≈7%) between 0°C and 144°C, with a peak around 144°C, likely due to the volatilization of aromatic and low‐molecular‐weight compounds naturally present in the extract [53]. Additional degradation events occur between 180°C and 420°C and around 560°C, corresponding to substantial mass losses (> 39% and > 34%, respectively), related to the thermal decomposition of organic constituents and carbonization of residual matter.

Regarding the polymeric films (Figure 8C), the first signs of thermal degradation were detected between 50°C and 100°C, likely resulting from the evaporation of residual solvents and volatile extract components. Similar to powdered chitosan and the extract, all films remained relatively stable within this range, showing less than 8% mass loss. Pronounced peaks between 130°C and 250°C are associated with degradation of the chitosan matrix, consistent with the significant mass loss observed in the TGA curves (Figure 8D), where all films lost approximately 59% of their initial mass.

The incorporation of increasing concentrations of MFAq:LCHA into the polymeric films did not significantly alter the temperatures corresponding to the main mass loss events when compared with the blank formulation (Film‐BL). However, a slight attenuation of the peaks between 50°C and 100°C and at approximately 206°C was observed for MFAq:LCHA‐0.5% and MFAq:LCHA‐1.0% films, suggesting minor stabilization effects. Conversely, the MFAq:LCHA‐2.5% film exhibited slight peak intensification, possibly resulting from polymer matrix disruption at higher extract loadings. Overall, the films showed a moderate reduction in thermal stability compared with powdered chitosan and the isolated extract, likely due to the formation of new intermolecular interactions and the higher moisture content associated with the film‐forming process.

#### 2.3.6 Fourier‐Transform Infrared Spectroscopy

Fourier‐transform infrared (FTIR) vibrational spectroscopy is a powerful analytical technique used to identify functional groups and investigate the structural backbone of compounds and matrices. In this study, FTIR was employed to explore possible intermolecular interactions between the MFAq and LCHA extracts and chitosan within the polymeric films. Each functional group absorbs infrared radiation at characteristic frequencies, allowing the identification of specific chemical bonds. Shifts or variations in band intensity can indicate molecular interactions, such as hydrogen bonding or electrostatic interactions, which alter the vibrational modes of functional groups [54].

In the spectra of the LCHA and MFAq extracts (Figure 9A), a broad band at 3290 cm^−1^ (LCHA) and a strong band at 3250 cm^−1^ (MFAq) correspond to [O–H] stretching vibrations of phenolic compounds [55]. Bands at 2920–2850 cm^−1^ (LCHA) and 2920–2880 cm^−1^ (MFAq) are attributed to [C–H] stretching of aliphatic groups. Both extracts display absorptions at 1390–1400 cm^−1^ (CH_3_) and a strong band near 1030 cm^−1^, characteristic of [C–OH] stretching in carboxylic acids [56]. In addition, LCHA exhibits distinct bands at 1690 and 1590 cm^−1^, assigned to [C═O] and [NH_2_] vibrations, while MFAq presents bands at 1600, 1350, 1260, and 1195 cm^−1^, corresponding to [C═C] aromatic, [C═O], [C–O], and [C–OH] stretching modes, respectively [57, 58].

**FIGURE 9:**
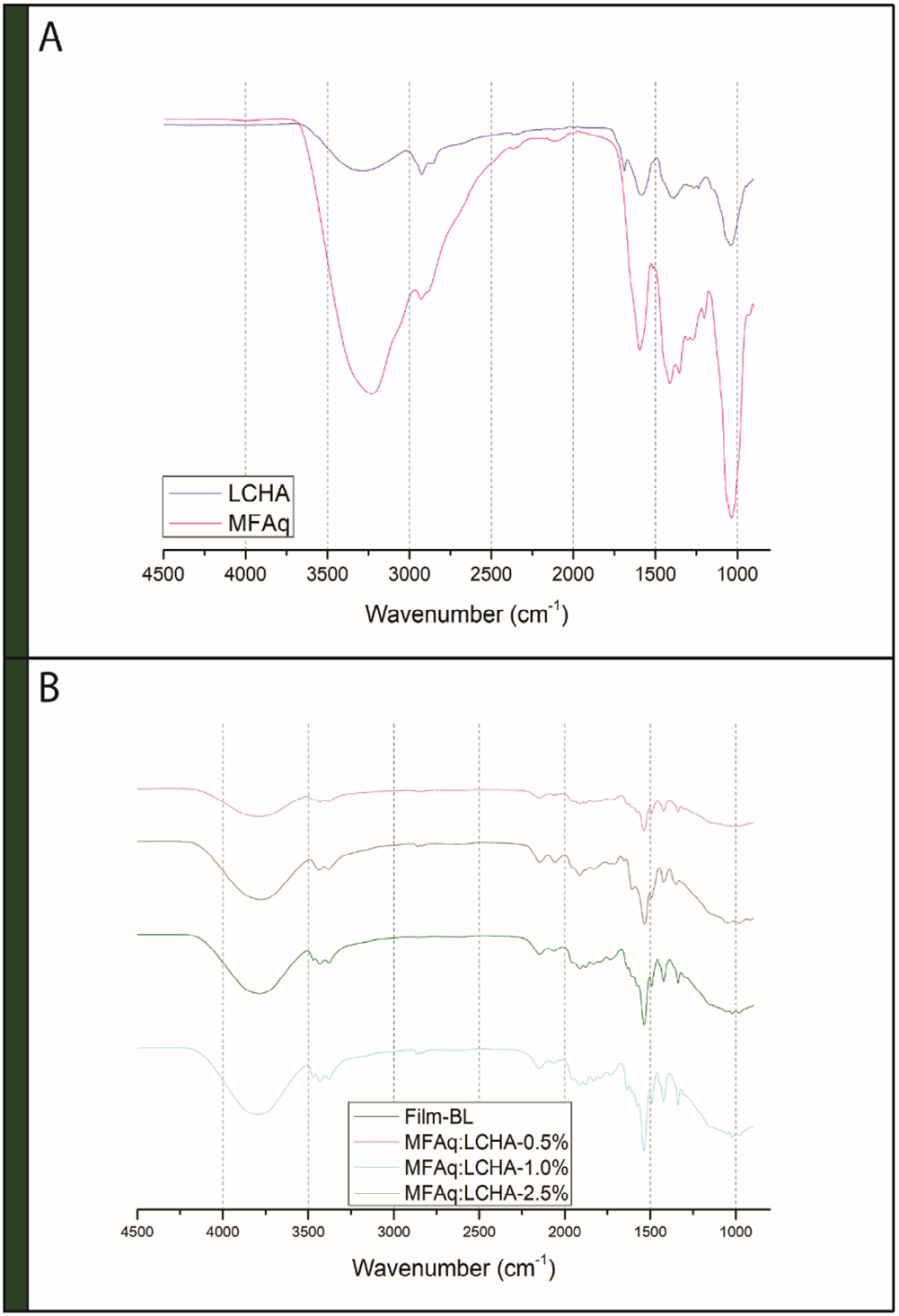
Overlaid spectra of MFAq and LCHA extracts (A) and chitosan films containing MFAq:LCHA associations (MFAq:LCHA‐0.5%–2.5%) and the blank film (Film‐BL) (B) obtained by Fourier Transform Infrared Spectroscopy (FTIR). FTIR spectra were acquired as the average of 64 consecutive scans using Perkin Elmer Spectrum Two software (Oxford Instruments), and data processing was performed using Origin 9.0 software.

The FTIR spectra of the polymeric films with and without MFAq:LCHA (Figure 9B) exhibit similar profiles, indicating comparable chemical compositions. A broad absorption between 3600 and 3000 cm^−1^ corresponds to overlapping [O–H] and [N–H] stretching vibrations of chitosan. The band at 1550 cm^−1^ is assigned to secondary amide [N–H] bending [59]. Films containing the MFAq:LCHA associations show an additional band near 3470 cm^−1^, associated with [N–H] and [O–H] stretching of amide derivatives, while other prominent bands (2870, 1590, 1530–1490, and 1420 cm^−1^) correspond to [CH_2_] of pyranose rings, [NH_2_], [NH], and [C–O] stretching, respectively [58, 60].

When comparing the spectra of films containing increasing concentrations of MFAq:LCHA, most bands remain consistent with those of the blank film (Film‐BL), except for notable variations at 3470, 1790, and 1020 cm^−1^, corresponding to [N–H], [C═O], and [C–OH] vibrations. These spectral differences suggest the occurrence of intermolecular interactions between chitosan and the extract components, likely involving hydrogen bonding or dipole‐dipole interactions. The observed changes in band intensity and position reflect the influence of extract concentration on the dipole moments of functional groups, confirming the establishment of molecular interactions within the polymeric matrix.

#### 2.3.7 Mechanical Properties

Mechanical strength is a critical parameter for topical formulations. In the case of films, they must withstand mechanical stress during application, handling, and transport, maintaining their integrity throughout the treatment period [53]. Dynamic mechanical analysis is also important to evaluate the influence of formulation components on the resistance and integrity of the films. In the axial tensile test (Figure 10A), failure due to stretching is assessed, whereas in the rotational torsion test (Figure 10B), film rupture under twisting is evaluated.

**FIGURE 10:**
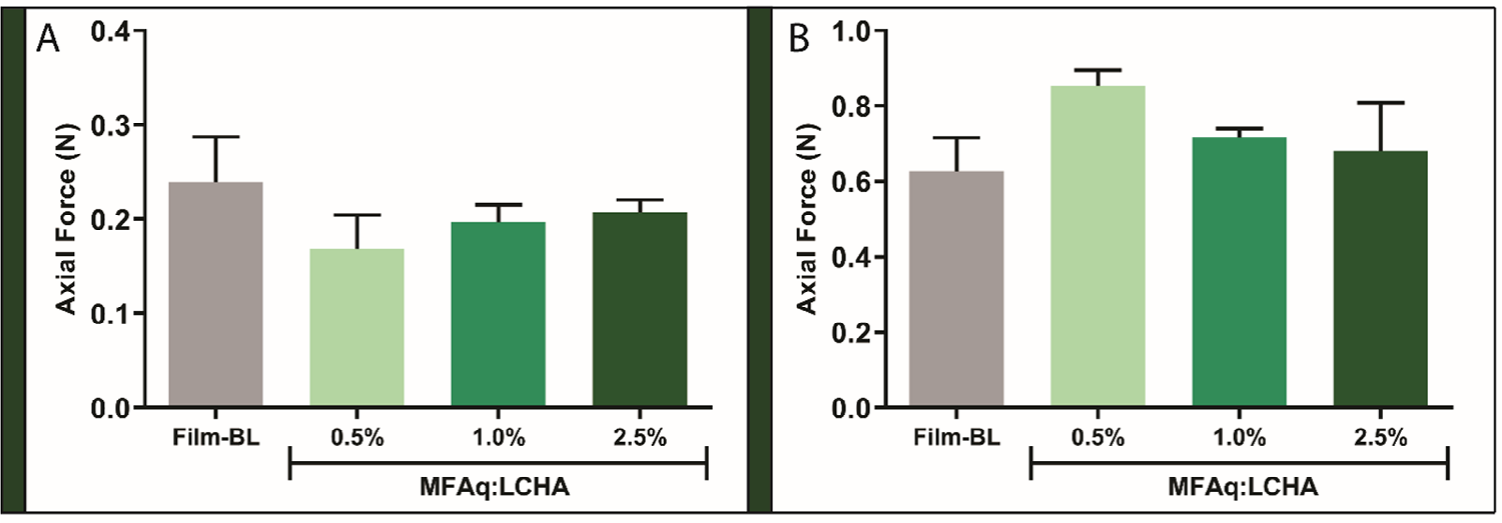
Rheological analysis of axial (A) and rotational (B) rupture from chitosan films containing the MFAq:LCHA associations (MFAq:LCHA‐0.5%–2.5%) and blank film (Film‐BL). Data are presented as mean ± standard deviation (SD) of quintuplicate measurements (*n* = 5). Statistical analysis was performed using one‐way ANOVA followed by Bonferroni's post hoc test. Differences were considered statistically significant at *p* < 0.05. Different letters indicate statistically significant differences between groups: a—different from Film‐BL; b—different from MFAq:LCHA‐0.5%; c—different from MFAq:LCHA‐1.0%; d—different from MFAq:LCHA‐2.5%.

The results from both axial and rotational rupture tests indicate that the incorporation of MFAq:LCHA did not significantly alter the films’ resistance to stretching or twisting compared to the blank film (Film‐BL) (*p <* 0.05). Whereas Fajardo et al. [3] and Silva et al. [39] reported a concentration‐dependent reduction in mechanical strength and elasticity, no statistical significance was observed among the MFAq:LCHA‐incorporated films in the present study. This suggests that the addition of the extract association does not affect film stiffness and allows a degree of extensibility regardless of extract content. Considering that wound‐healing films should exhibit high tensile strength for application and handling, these results are satisfactory and demonstrate the practical applicability of the developed formulation [3].

### 2.4 Biological Properties

#### 2.4.1 Cell Viability

As performed for the extract associations, the cytotoxicity of the polymeric films was also assessed using the MTT assay in peritoneal macrophage cultures and L929 fibroblast cell lines, with results expressed as percentage of cell viability. This assay is essential to evaluate the safety of the films for potential human use, since macrophages are immune cells directly involved in the inflammatory process, while fibroblasts are mesenchymal cells responsible for synthesizing collagen and other macromolecules, playing a central role in tissue repair and chronic inflammation [61]. Therefore, assessing the cytotoxicity of the samples against these cell types is crucial, considering the intended topical application, which justified their selection for this assay.

According to NBR ISO 10993‐5 [17], both macrophages (Figure 11A) and fibroblasts (Figure 11B) maintained satisfactory viability after treatment with all polymeric films, preserving at least 70% cell viability compared with the negative control (DMSO) after 48 h of exposure. These results indicate that the films are suitable for application, as no evidence of cytotoxicity was observed, which is consistent with the literature describing chitosan as a biocompatible material. This finding also aligns with the initial analysis previously performed and discussed for the associations, including MFAq:LCHA (1:1) (in Figure 1), which did not exhibit cytotoxicity when compared with the negative control, with no viability values falling below 70% in assays using peritoneal macrophages.

**FIGURE 11:**
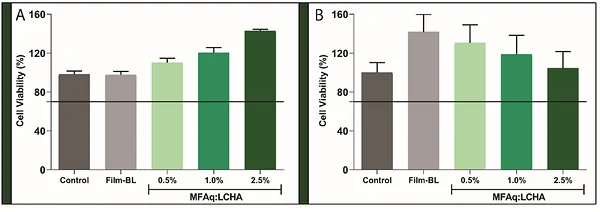
Assessment of the peritoneal macrophages (A) and L929 fibroblasts (B) viability after treatment with chitosan films containing MFAq:LCHA associations (MFAq:LCHA‐0.5%–2.5%) and blank film (Film‐BL). Data are presented as mean ± standard deviation (SD) of two independent experiments performed in triplicate using 0.2 × 0.2 cm film fragments (*n* = 6). Statistical analysis was performed using one‐way ANOVA followed by Bonferroni's post hoc test. Differences were considered statistically significant at *p* < 0.05. a—statistically different from the control group. The horizontal line indicates the minimum viability threshold of 70%.

#### 2.4.2 Anti‐Inflammatory Activity

As performed for MFAq:LCHA, NO production was quantified to evaluate the anti‐inflammatory activity of the polymeric films (Figure 12A). A dose‐response effect was observed, with Film‐BL showing a reduction of 19.48% ± 0.73%, MFAq:LCHA‐0.5% a reduction of 24.66% ± 3.65%, MFAq:LCHA‐1.0% approximately 42.42% ± 3.95%, and MFAq:LCHA‐2.5% a reduction of 59.58% ± 3.29%. All films demonstrated a significant reduction compared with the control group (*p* < 0.05). When comparing films with each other, only MFAq:LCHA‐2.5% showed a statistically significant difference relative to the blank film, while MFAq:LCHA‐1.0% exhibited results comparable to MFAq:LCHA‐2.5%.

**FIGURE 12:**
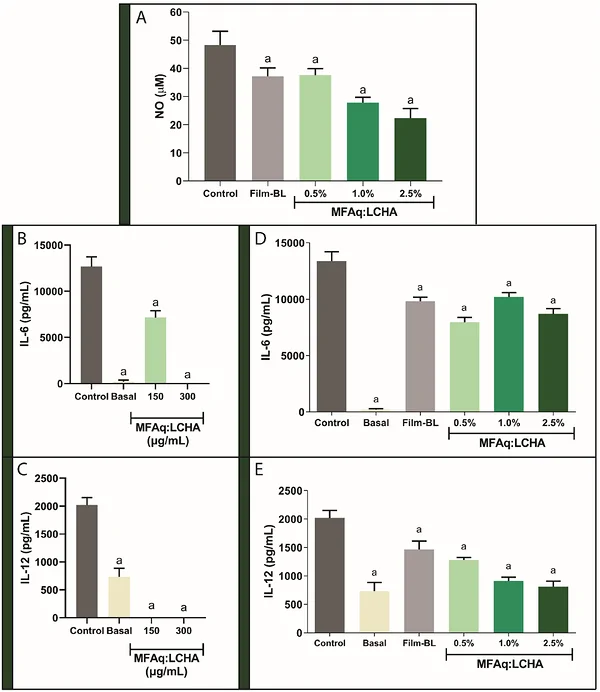
Anti‐inflammatory activity from MFAq:LCHA (B‐C) and chitosan films containing MFAq:LCHA associations (MFAq:LCHA‐0.5‐2.5%) and blank film (Film‐BL) (A, D–E). Nitric oxide (A), IL‐6 (B–D) and IL‐12 (C–E) cellular production after treatment. Data are presented as mean ± standard deviation (SD) of two independent experiments performed in triplicate using 0.2 × 0.2 cm film fragments (*n* = 6). Statistical analysis was performed using one‐way ANOVA followed by Bonferroni's post hoc test. Differences were considered statistically significant at *p* < 0.05. a—statistically different from the control group; b—statistically different from the basal group; c—statistically different from Film‐BL.

In addition to NO quantification, the levels of cytokines IL‐6 and IL‐12 were measured to further elucidate the anti‐inflammatory mechanism of the films and MFAq:LCHA association 1:1 (for comparison). Cytokines are key modulators of the immune system and inflammation, and their regulation is crucial in the treatment of various pathologies and disorders. Specifically, IL‐6 and IL‐12 play central roles in immune and inflammatory processes [62]. IL‐6 regulates complex cellular processes such as proliferation and differentiation, and its dysregulated signaling and impaired cellular communication are associated with severe diseases, including cancer, autoimmune disorders, and chronic inflammatory conditions [63]. Conversely, IL‐12 is secreted by activated antigen‐presenting cells (APCs), including dendritic cells, monocytes, and macrophages. Its activation promotes the proliferation and differentiation of T helper 1 (Th1) cells and stimulates interferon‐gamma (IFN‐γ) production, thus playing a pivotal role in immune responses [62].

Analyzing first the MFAq:LCHA association 1:1, it significantly reduced cytokine levels (*p* < 0.05) at both evaluated concentrations. In the analysis of IL‐6 (Figure 12B), while the baseline condition exhibited a 98.41% reduction in cytokine levels (compared to the control), MFAq:LCHA achieved a superior outcome at 300 µg/mL, with a 100% reduction, and a reduction of 43.51% at 150 µg/mL. For IL‐12 (Figure 12C), the association showed a higher percentage of reduction than the baseline (94.52%) at both concentrations, reaching 100% reduction in cytokine levels at 150 and 300 µg/mL. These findings reinforce the anti‐inflammatory potential of the association.

Meanwhile, all films showed significant reduction for IL‐6 (Figure 12D) and IL‐12 (Figure 12E) levels compared with the control group (*p* < 0.05). Regarding IL‐6, reductions were 26.64% ± 2.36%, 40.57% ± 3.48%, 26.64% ± 1.35%, and 35.01% ± 3.02% for Film‐BL, MFAq:LCHA‐0.5, MFAq:LCHA‐1.0%, and MFAq:LCHA‐2.5%, respectively. For IL‐12, films MFAq:LCHA‐1.0% (56.01% ± 2.87%) and MFAq:LCHA‐2.5% (62.27% ± 0.50%) achieved the highest reductions, while Film‐BL and MFAq:LCHA‐0.5% reduced 24.32% ± 2.38% and 37.56% ± 1.75%. When films containing extracts were compared with the blank film, both MFAq:LCHA‐1.0% and MFAq:LCHA‐2.5% displayed statistically superior reductions in IL‐12 levels (*p* < 0.05). These findings demonstrate a dose‐dependent effect, particularly for films containing higher MFAq:LCHA concentrations.

Similar to nitric oxide, pro‐inflammatory cytokines play a relevant role in tissue repair; however, their excessive production can impair wound recovery [64]. Therefore, modulating cytokine production is critical for ensuring proper healing. The present results highlight the potential of the formulations to reduce inflammatory mediators. This activity may be partly explained by the intrinsic bioactivity of chitosan, as evidenced by the blank film results, and further enhanced by the incorporation of MFAq:LCHA extracts, suggesting synergistic interactions between the phytochemicals and the polymeric matrix that potentiate the anti‐inflammatory response.

The chemical characterization of MFAq:LCHA revealed six terpenes, five flavonoids, including quercetin derivatives such as quercetin‐hexosyl‐pentoside and quercetin‐hexoside, and one azanthraquinone. Terpenes are reported to possess anti‐inflammatory properties by decreasing IL‐6 and TNF‐α production in mast cells, inhibiting LTC4 (leukotriene C4 synthase) release, and modulating TXB2 (thromboxane B2) release. Their general effect involves downregulation of pro‐inflammatory cytokine expression and NO production through multiple molecular targets, including cytokines, transcription factors, autophagy machinery, membrane receptors, and other mediators, enabling them to act simultaneously on distinct signaling pathways [28]. Quercetin and its derivatives are well described for their potent anti‐inflammatory effects, capable of reducing both acute and chronic inflammatory processes [29]. Additionally, azanthraquinones have been associated with the inhibition of NO production, downregulation of iNOS and COX‐2 expression, and suppression of PGE2 release in macrophages [30].

The anti‐inflammatory findings, together with the release study data, demonstrate that the films are capable of delivering phytoconstituents with biological activity. This effect is likely attributable to phenolic compounds and terpenes, which are present in high concentrations in the extracts. Moreover, films containing 1.0% and 2.5% MFAq:LCHA exhibited the most pronounced effects, particularly in the inhibition of NO and cytokine production. These outcomes are consistent with previous findings, especially for MFAq:LCHA‐1.0%, which displayed greater physicochemical stability compared with MFAq:LCHA‐2.5%.

#### 2.4.3 Antioxidant Activity

To evaluate the antioxidant activity of the polymeric films, two assays were employed: the DPPH radical scavenging method and the total antioxidant capacity (TAC) by the phosphomolybdenum assay.

Regarding the DPPH results (Figure 13A), the films exhibited a clear dose‐dependent response. The formulation with the highest concentration of the MFAq:LCHA association (MFAq:LCHA‐2.5%) demonstrated the strongest radical scavenging capacity (91.34% ± 2.64%), whereas MFAq:LCHA‐1.0% (56.70% ± 2.64%) and MFAq:LCHA‐0.5% (34.19% ± 2.44%) showed proportionally lower activities. The blank film, Film‐BL, displayed a scavenging activity of 8.22% ± 1.73%, consistent with literature that reports that chitosan possesses intrinsic antioxidant activity. Notably, no statistically significant difference (*p* < 0.05) was observed between MFAq:LCHA‐2.5% and the standard rutin. These results indicate that the antioxidant activity of the formulations is directly proportional to the MFAq:LCHA concentration incorporated into the films, confirming a dose‐dependent effect consistent with the findings for MFAq:LCHA (1:1).

**FIGURE 13:**
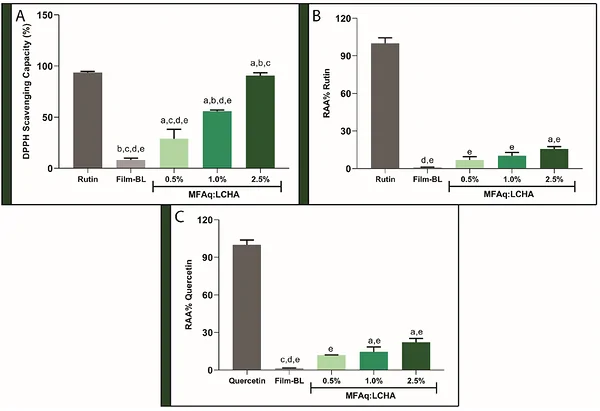
Antioxidant activity from chitosan films containing MFAq:LCHA associations (MFAq:LCHA‐0.5%–2.5%) and blank film (Film‐BL). DPPH scavenging method (A) and total antioxidant capacity relative to rutin (B) and quercetin (C). Data are presented as mean ± standard deviation (SD) of two independent experiments performed in triplicate using 0.4 × 0.4 cm film fragments (*n* = 6). Statistical analysis was performed using one‐way ANOVA followed by Bonferroni's post hoc test. Differences were considered statistically significant at *p* < 0.05. a—statistically different from Film‐BL; b—statistically different from MFAq:LCHA‐0.5%; c—statistically different from MFAq:LCHA‐1.0%; d—statistically different from MFAq:LCHA‐2.5%; e—statistically different from rutin; f—statistically different from quercetin.

For the phosphomolybdenum reduction assay, the relative antioxidant activity (RAA%) of the films in comparison with rutin (Figure 13B) and quercetin (Figure 13C) also followed a dose‐dependent trend. The film containing the highest extract concentration, MFAq:LCHA‐2.5%, presented the greatest activity (*p* < 0.05). When compared to rutin, the RAA% values were 1.03 ± 0.08% for Film‐BL, 6.79% ± 2.65% for MFAq:LCHA‐0.5%, 10.21% ± 2.70% for MFAq:LCHA‐1.0%, and 15.57% ± 2.04% for MFAq:LCHA‐2.5%. Against quercetin, the results were 1.47% ± 0.12%, 11.91% ± 0.18%, 14.60% ± 3.87%, and 22.27% ± 2.92%, respectively. As observed in the DPPH assay, the antioxidant activity was directly proportional to the MFAq:LCHA concentration in the formulations.

As previously reported, the phytochemical characterization of MFAq:LCHA revealed the presence of terpenes, flavonoids, and an azanthraquinone, all which likely contribute to the observed antioxidant profile. Terpenes have been reported to exert antioxidant and photoprotective effects by inhibiting UVB‐induced free radical production. Their mechanisms of action involve reduction of H2O2‐induced lipid peroxidation, suppression of ROS and superoxide generation, and stimulation of enzymatic antioxidant defenses such as catalase, superoxide dismutase, and peroxidases. Flavonoids, in turn, are well‐known ROS scavengers, with their high degree of hydroxylation being critical for their radical‐neutralizing capacity and overall antioxidant efficacy [7, 29].

Overall, the antioxidant results are consistent with those obtained for the extract associations, particularly MFAq:LCHA (1:1), which showed radical scavenging activity in a dose‐dependent manner, likely attributable to its high levels of phenolic compounds and terpenes, such as rutin and ursolic acid, which were identified by UFLC‐QTOF‐MS. Among the tested films, MFAq:LCHA‐2.5% stood out, showing a radical scavenging capacity comparable to rutin; however, MFAq:LCHA‐1.0% also presented relevant activity.

Kadam et al. [65] evaluated the antioxidant activity of a chitosan film incorporated with *Cedrus deodara* extract and similarly reported increasing antioxidant activity proportional to extract concentration. These findings corroborate the present results, reinforcing that the films are capable of releasing phytoconstituents from their matrix.

Taken together, the antioxidant data and release study findings indicate that the polymeric films can effectively deliver bioactive phytoconstituents with antioxidant properties, which can be attributed to the high levels of phenolic compounds and terpenes in the extracts.

#### 2.4.4 Wound Healing Activity

Wound healing is a process that restores the integrity of injured tissue and is characterized by the phases of inflammation, proliferation, and maturation. These stages promote tissue reconstruction through the reorganization of a new extracellular matrix, ultimately leading to healing [66]. Accordingly, the wound‐healing activity of the polymeric films was investigated using the scratch assay. Images were obtained at 0, 24, and 48 h, allowing evaluation of cell migration by fluorescence microscopy software and calculation of the migration rate based on the reduction of the scratch area over time.

Initially, the assay was conducted using the MFAq:LCHA association 1:1 for comparative purposes. As shown in Figure 14, within the first 24 h, the association at both tested concentrations significantly promoted cell migration, reaching 79.64% ± 1.56% and 88.63% ± 2.24%. After 48 h of treatment, a further increase was observed (90.08% ± 5.40% and 90.33% ± 2.08%, respectively). These results indicate that the association effectively enhances the wound healing process, as significantly higher outcomes were obtained compared to the control group, with stimulation being directly proportional to the tested concentration (*p* < 0.05).

**FIGURE 14:**
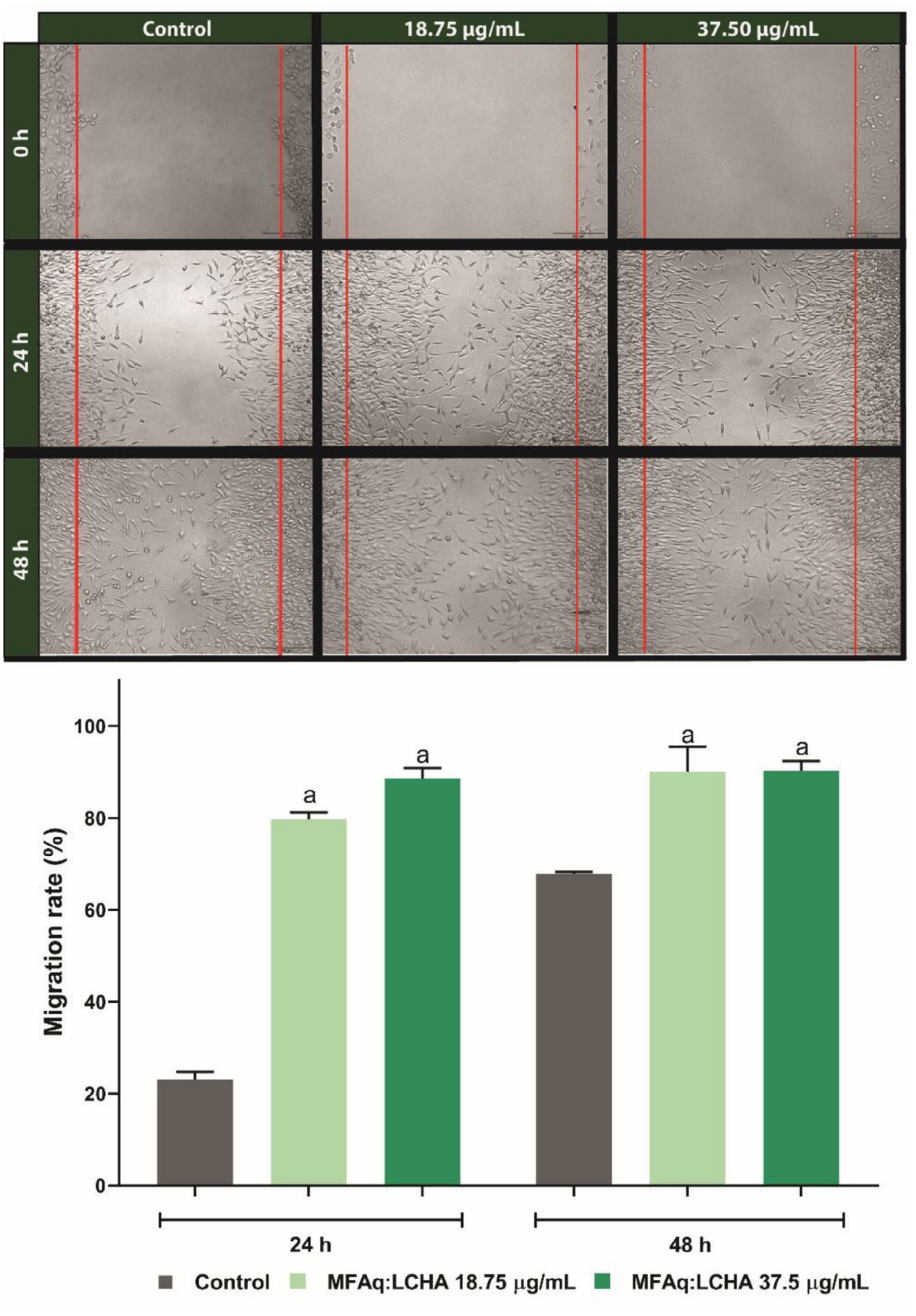
Wound healing capacity of MFAq:LCHA association at concentrations ranging from 18.75 to 37.50 µg/mL. Data are presented as mean ± standard deviation (SD) of two independent experiments performed in triplicate (*n* = 6). Statistical analysis was performed using one‐way ANOVA followed by Bonferroni's post hoc test. Differences were considered statistically significant at *p* < 0.05. a—statistically different from the control group.

For the films, as shown in Figure 15, during the first 24 h, films MFAq:LCHA‐2.5%, MFAq:LCHA‐1.0%, and MFAq:LCHA‐0.5% significantly stimulated fibroblast migration by 76.77% ± 6.24%, 74.13% ± 5.00%, and 17.67% ± 4.29%, respectively. After 48 h of treatment, this effect further increased, with migration rates of 96.83% ± 2.20%, 95.68% ± 1.80%, and 81.34% ± 5.38%, respectively, including fibroblasts treated with Film‐BL (70.11% ± 3.39%). These findings indicate that the films effectively promote cutaneous repair after injury, as they were significantly superior to the untreated control group. Fibroblast migration was directly proportional to extract concentration. However, no significant differences were found between MFAq:LCHA‐1.0% and MFAq:LCHA‐2.5%, although both exhibited statistically higher wound‐healing potential compared with Film‐BL (*p* < 0.05).

**FIGURE 15:**
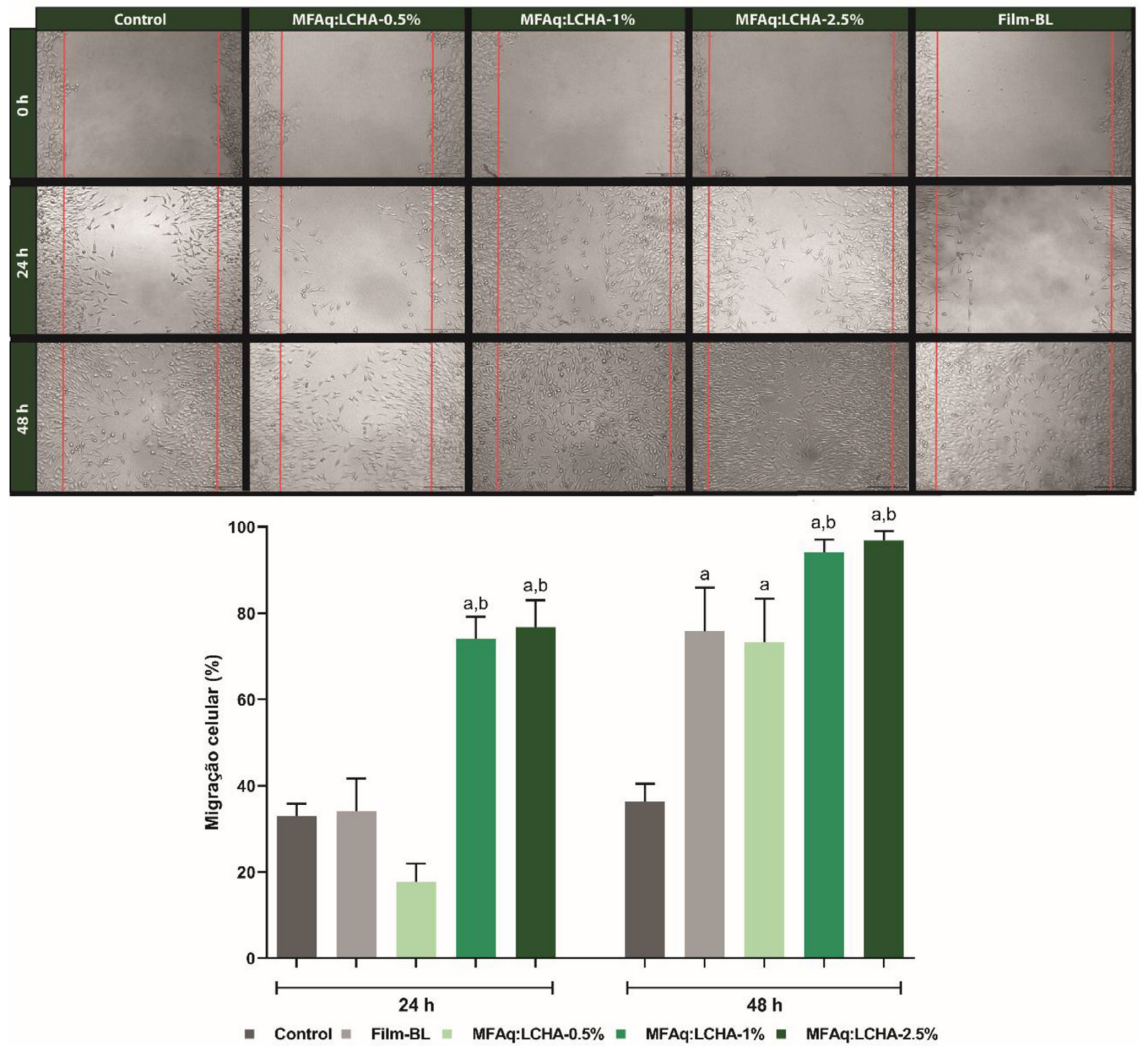
Wound healing capacity from chitosan films containing the MFAq:LCHA associations (MFAq:LCHA‐0.5%–2.5%) and blank film (Film‐BL). Data are presented as mean ± standard deviation (SD) of two independent experiments performed in triplicate using 0.1 × 0.1 cm film fragments (*n* = 6). Statistical analysis was performed using one‐way ANOVA followed by Bonferroni's post hoc test. Differences were considered statistically significant at *p* < 0.05. a—statistically different from the control group; b—statistically different from Film‐BL.

When comparing the results obtained for MFAq:LCHA with those of the polymeric films, an increase in activity was observed, with higher cell migration rates for MFAq:LCHA‐1% (95.68% ± 1.80%) and MFAq:LCHA‐2.5% (96.83% ± 2.20%) after 48 h of treatment, compared with the association at both tested concentrations (90.08% ± 5.40% and 90.33% ± 2.08%). This wound‐healing activity may be attributed to chitosan, which has been reported to stimulate fibroblast proliferation and angiogenesis, modulate collagen deposition, and enhance hyaluronic acid synthesis at the lesion site, thereby accelerating wound closure and minimizing scar formation [4].

The potent activity observed for MFAq:LCHA may also be related to its phytochemical profile. Terpenes, which were detected in the association, are known for their low‐toxicity profile and stimulatory effect on fibroblast growth. Mechanistically, these compounds can modulate transforming growth factor (TGF‐β1) signaling and type I procollagen secretion, processes essential for extracellular matrix remodeling during the proliferative phase of healing [28]. Furthermore, two quercetin derivatives (quercetin‐hexosyl‐pentoside and quercetin‐hexoside) were identified in MFAq:LCHA, and quercetin itself has been reported to accelerate wound repair by reducing skin lesion area, improving tissue viscoelasticity, alleviating pain, and facilitating recovery in chronic wounds [29]. These mechanisms may synergize with chitosan's bioactivity, contributing to the enhanced wound closure observed.

Although the wound‐healing potential of *M. frigidus* has not been previously reported, extracts from *L. camara* leaves have demonstrated dose‐dependent wound‐healing activity in infected wounds and burn injuries [67]. Thus, the combination of chitosan, with well‐documented healing activity, and MFAq:LCHA, supported by literature reports for *L. camara*, may explain the observed effect and its enhancement upon incorporation of the plant extracts into the formulation. Together with the release study results, these findings suggest that the films enable the controlled release of bioactive phytochemicals from MFAq and LCHA, which appear to act synergistically with chitosan in the in vitro wound‐healing process.

Notably, MFAq:LCHA‐1.0% and MFAq:LCHA‐2.5% films showed the most pronounced effect, achieving nearly complete wound closure (≈100%) after 48 h of treatment and demonstrating statistically significant differences compared with Film‐BL. Consistent with the results of anti‐inflammatory and antioxidant evaluations, the release capacity of the films, coupled with their rheological and physicochemical characteristics, highlights the wound‐healing potential of the formulation, particularly at concentrations of 1.0% and 2.5%.

## 3 Conclusion

This study demonstrated that the association of *Mitracarpus frigidus* and *Lantana camara* extracts in a 1:1 ratio presents a balanced phytochemical profile, low cytotoxicity, and significant antioxidant and anti‐inflammatory activities. Incorporation of this association into chitosan films resulted in formulations with suitable physicochemical, rheological, and thermal properties for topical application. Among the tested formulations, films containing 1.0% and 2.5% of the extract association showed the most pronounced biological effects, including inhibition of nitric oxide and pro‐inflammatory cytokines, strong antioxidant activity, and enhanced fibroblast migration in vitro. Overall, these findings demonstrate that MFAq:LCHA‐loaded chitosan films represent a promising and sustainable strategy for the treatment of skin disorders associated with inflammation and oxidative stress. While in vitro results are highly compelling, further in vivo studies are warranted to confirm their therapeutic efficacy and safety.

## 4 Experimental Section

### 4.1 Plant Material

*Mitracarpus frigidus* aerial parts and *Lantana camara* leaves were collected in Juiz de Fora, Minas Gerais, Brazil. The *M. frigidus* sample (coordinates: 21°41′20″′ S, 43°20′40′′ W) was collected in May 2022, and the *L. camara* sample (coordinates: 21°77′62′′ S, 43°37′05′′ W) was collected in January 2023. The botanical identification of the species was provided by Dr. Vinícius Antônio de Oliveira, and each was deposited in the Leopoldo Krieger Herbarium of the Federal University of Juiz de Fora (CESJ 46076 and CESJ 30671, respectively), under license numbers A032F41‐23 and A18AB08 SISGEN/BRASIL.

### 4.2 Reagents and Materials

Chitosan (low molecular weight: 50.000–190.000 Da, degree of deacetylation ≥ 75%, solubility: dilute aqueous acid, soluble), quercetin, rutin, lipopolysaccharide (LPS), IFN‐γ, 2,2‐diphenyl‐1‐picrylhydrazyl (DPPH), 3‐(4,5‐Dimethyl‐2‐thiazolyl)‐2,5‐diphenyl‐2H‐tetrazolium bromide (MTT) and N‐(1‐Naphthyl) ethylenediamine Dihydrochloride (NED) were purchased from Sigma‐Aldrich Co. Ltd., USA. Dulbecco's Modified Eagle Medium (DMEM), Roswell Park Memorial Institute (RPMI), and fetal bovine serum (FBS) were obtained from Gibco, USA. Glycerin, ethanol, methanol, and acetic acid were obtained from Cinord Indústria Farmacêutica Ltda (Brazil), and Dinâmica Química Contemporânea Ltda (Brazil), Isofar Indústria e Comércio de Produtos Químicos Ltda (Brazil), respectively. Both NaCl and KCl were provided by Merck S/A, Germany. NaHCO_3_ was purchased from Quimibras Indústrias Químicas S/A (Brazil). Sulfanilamide and NaH_2_PO_4_ were provided by Vetec Química Fina Ltda (Brazil). Folin‐Ciocalteu reagent, dimethyl sulfoxide (DMSO), and ammonium molybdate were obtained from BP Biomedicals (USA) and Biotec Reagentes Analíticos (Brazil), respectively.

### 4.3 Extracts Preparation

The *M. frigidus* extract (MFAq) was prepared by infusing 1 kg of dried and powdered aerial plant parts in distilled water at 80°C for 2 h. Following infusion, the solution was filtered and lyophilized using a Martin Christ Alpha 1‐2 LDplus freeze dryer, yielding a dry, powdered extract. This process resulted in a 12.6% yield relative to the initial plant material, which is a key indicator for assessing the effectiveness and reproducibility of the extraction method.

*L. camara* leaves (135.98 g), after being dried and powdered, underwent exhaustive maceration using a 1:1 mixture of distilled water and ethyl alcohol. The maceration process was performed in three cycles of 24 h each, with mild heating and ultrasonic bath assistance to enhance extraction efficiency. The resulting liquid extract was concentrated with a BUCHI Labortechnik AG (V‐700) rotary evaporator under reduced pressure and a water bath (45°C–55°C). Subsequent lyophilization (Martin Christ Alpha 1‐2 LDplus) removed any residual solvent. This extract, designated LCHA, was then weighed, yielding 10.28%.

### 4.4 Preparation of MFAq:Lcha Associations

MFAq:LCHA associations were prepared at various ratios. The extracts were mixed based on their dry weight to ensure standardized concentrations. Five associations were created: MFAq:LCHA at 20:80 (1:4), 40:60 (2:3), 60:40 (3:2), 80:20 (4:1), and 50:50 (1:1). These ratios were chosen to provide a gradual 20% variation in extract composition, as well as an equal proportion (50:50), allowing for the evaluation of potential synergistic or antagonistic effects between the extracts.

### 4.5 Phytochemical Analysis

#### 4.5.1 Total Phenolic Content

The total phenolic content was determined using the Folin‐Ciocalteu method [68]. A calibration curve was generated using tannic acid as the standard. A stock solution of the MFAq:LCHA associations (1 mg/mL) was prepared in ethanol. Absorbance was measured at 770 nm using a Thermo Scientific Multiskan GO UV‐Vis spectrophotometer with software version 3.2. The phenolic compound content was expressed as µg of tannic acid equivalents per mg of plant extract. Three independent experiments were performed, both in triplicate.

#### 4.5.2 Terpenes Content

The total terpene content was quantified using the colorimetric method described by Pedrosa et al. [69]. Lupeol, a triterpene, was used to create the calibration curve. A stock solution of the MFAq:LCHA associations was prepared in ethanol at 1 mg/mL. Absorbance was measured at 548 nm using a Thermo Scientific Multiskan GO UV‐Vis spectrophotometer with software version 3.2. The results, expressed as µg of lupeol equivalent per mg of plant extract, were derived from three independent experiments, each performed in triplicate.

#### 4.5.3 UFLC‐QTOF‐MS Analysis

The most promising MFAq:LCHA association, which demonstrated superior chemical and biological properties, was analyzed by UFLC‐QTOF‐MS in positive mode. This method involved scanning ions in the m/z range of 100–1200. The mobile phase consisted of water (A) and acetonitrile (B), both of which were acidified with 0.1% formic acid. The gradient was as follows: 0–4 min: 5% B; 4–34 min: ramp to 100% B; 34–38 min: held at 100% B; then returned to initial conditions (5% B) over 8 min, with an additional 4 min for re‐equilibration. The injection volume was 3.0 µL at a concentration of 1 mg/mL diluted in HPLC‐grade methanol, the flow rate was 0.5 mL/min, and the run time was 50 min. Analysis was performed using a C18 reverse‐phase column (4.6 × 150 mm, 2.6 µm) maintained at 40°C. Electrospray ionization (ESI) was used for ionization, with a capillary voltage of 3.5 kV and a temperature of 325°C. Fragmentation profiles were compared with literature data to identify sample constituents. This analysis was conducted in collaboration with Fiocruz using the Analytical Methods Platform at the Federal University of Rio Grande do Norte.

### 4.6 Preparation of Films Containing MFAq:LCHA Association

The films were prepared using the solvent evaporation technique, as described by Escárcega et al. [40]. Only the most promising MFAq:LCHA association, demonstrating superior chemical and biological properties, was chosen for the formulation. Initially, a 2% (w/v) chitosan (low molecular weight and degree of deacetylation ≥ 75%, Sigma‐Aldrich) solution was prepared in 1% (v/v) acetic acid under mechanical stirring (Fisatom) at approximately 1100 rpm for 1 h to ensure complete protonation and dissolution of the polymer. Subsequently, the plasticizer (7.5% v/v glycerol) was added under continuous stirring for an additional 30 min. The pH of the solution was measured using a pH meter and adjusted to a skin‐compatible range (4.6 to 5.8) with 2.5% (v/v) NaOH. Next, the solution was divided into four 12 mL aliquots containing the extract at concentrations of 0.5% (w/v), 1% (w/v), 2.5% (w/v), and a control (without sample). The samples were weighed and solubilized in 2 mL of distilled water and 0.6 mL of ethanol, then incorporated into the respective precursor solution fractions. For the blank, only 2 mL of distilled water and 0.6 mL of ethanol were incorporated. The mixtures were degassed and further solubilized in an ultrasonic bath for 1 h at room temperature. Finally, the aliquots were cast into 9 cm diameter Petri dishes (63.615 cm^2^) and dried in an oven at 45°C for 24 h. Each film exhibited final concentrations of 38.11, 96.79, and 202.83 mg/cm^3^, corresponding to the 0.5%, 1.0%, and 2.5% formulations, respectively. The films were stored under refrigeration at 5°C.

### 4.7 Characterization of Film Precursor Solutions

#### 4.7.1 Zeta Potential and Electrical Conductivity

Electrical conductivity (EC) and zeta potential (ZP) of the precursor solutions were performed using a Zetasizer Nano ZS90 particle analyzer (Malvern Instruments), employing the Doppler micro‐electrophoresis technique. Measurements were conducted at a light scattering angle of 173° and an applied alternating electric field of 10 V. ZP values were calculated based on electrophoretic mobility using the Smoluchowski model. Samples were loaded into a DPS1070 disposable polyethylene capillary cell equipped with electrodes, and the results were expressed as the mean of five independent measurements for each sample. Data analysis was carried out using GraphPad Prism 8.0.1 software [70].

#### 4.7.2 Determination of Particle Size Distribution Using Dynamic Light Scattering (DLS)

Particle size determination was performed using a Zetasizer Nano ZS90 (Malvern Instruments), equipped with a photometric module for dynamic light scattering (DLS) measurements. At a controlled temperature of 25°C, 100 µL of each sample was diluted in 1.9 mL of deionized water and transferred to a 1 cm pathlength disposable polyethylene cuvette. The samples were then exposed to a monochromatic light beam (He‐Ne laser, 4 mW, 633 nm), and scattered light was detected at an angle of 90°. Ten independent measurements were performed, with ten runs per measurement. Final particle size values were expressed as the mean of these replicates. Data analysis was carried out using GraphPad Prism 8.0.1 software [70].

#### 4.7.3 Rheological Parameters

The rheological behavior of the precursor solutions was evaluated using steady‐state and oscillatory rheology on an automated DHR‐1 rheometer (TA Instruments). Flow and viscosity curves as a function of shear rate were obtained under isothermal conditions, while viscosity‐versus‐temperature profiles were recorded under isorotational conditions. The viscoelastic properties of the materials were also assessed through amplitude sweep tests, in which the loss modulus (*G*'') and storage modulus (*G*') were plotted against shear stress. All measurements were performed using a plate‐plate geometry with a gap of 1000 µm at a controlled temperature of 25°C via Peltier plate and shear rates ranging from 0 to 1000 s^−1^. Temperature‐dependent experiments were carried out by cyclically ramping the temperature from 5°C to 45°C at a constant shear rate of 5 s^−1^. Data processing and analysis were conducted using Origin 9.0 software [70].

### 4.8 Characterization of Films

#### 4.8.1 In Vitro Drug Release Experimentation

To evaluate the phytoconstituent release from the polymeric films, a study in a unidirectional diffusion device was conducted. The films were fixed on the donor compartment, while the receptor compartment was filled with simulated wound exudate (composed of 0.68 g NaCl, 0.22 g KCl, 2.5 g NaHCO_3_, and 0.35 g NaH_2_PO_4_ in 100 mL of distilled water at pH 7.4, as described by Pansara et al. [71]). The system was kept under constant stirring at a controlled temperature range of 32°C–34°C and at sink condition. Samples were collected at predetermined time intervals and replaced with an equivalent volume of simulated wound exudate for a total of 8 h of experimentation. The absorbance was measured at 270 nm (Thermo‐Scientific SkanIt Multiskan GO, software 3.2). A standard curve of MFAq:LCHA association was constructed by dissolving predetermined concentrations of the association in simulated wound exudate at concentrations ranging from 0.0037 to 0.0250 mg/mL, and the absorbance was measured at 270 nm (*R* ^2^ = 0.9897). The experiment was performed in triplicate.

#### 4.8.2 Morphology

The morphological characterization of the microstructures of the films were performed using scanning electron microscopy (SEM) (JEOL JSM‐6390LV, Tokyo, Japan). The samples (0.5 × 0.5 cm squares) were mounted on aluminum stubs using carbon adhesive tape and analyzed under high vacuum conditions. Surface morphology was examined at magnifications of 500× and 3500×, while cross‐sectional analysis was conducted at magnifications ranging from 400× to 800×.

#### 4.8.3 Weight, Thickness, and Moisture Content

Average weight, thickness, and moisture content of the films was performed according to the methodology proposed by Shah et al. [46]. Ten 1 × 1 cm squares of each film were weighed using an analytical balance (Shimadzu AY220), and their thickness was measured using a portable digital micrometer (Insize, model 3109‐25), with 0.001 mm precision over a range of 0‐25 mm. All measurements were carried out in quadruplicate, and results were expressed as mean ± standard deviation. The moisture content was determined gravimetrically, with four 1 × 1 cm film sections being weighed (initial weight, Pi) on an analytical balance and then placed in a forced‐air oven (Quimis, model Q316M2) at 60°C for 24 h. After this period, the final weight (Pf) was recorded. Analyses were performed in quadruplicate, and the moisture content (%) was calculated using the following equation:

(1) $$Moisture\left(\%\right)=\frac{W_{i}-W_{f}}{W_{i}}\times 100$$

where *W*_i_ means the initial weight of the film; *W*_f_ means the final weight after drying.

#### 4.8.4 Swelling Ratio and Solubility Degree

The swelling degree of the films was determined according to the methodology described by Pansara et al. [71]. Film samples measuring 1 × 1 cm were previously weighed (W0) using an analytical balance (Shimadzu AY220) and then immersed in simulated wound fluid at 32°C. At predetermined time intervals the films were removed, gently blotted with filter paper to eliminate excess fluid, and weighed again (*W*_i_). At the end, the same films were used to determine the solubility degree, following the method described by Escárcega et al. [40]. The swollen films were dried in an oven for an additional 24 h and weighed again (*W*_f_). All analyses were conducted in quadruplicate, and the swelling capacity (Swelling %) and solubility percentage (Solubility %) were calculated using the equation:

(2) $$Swelling\left(\%\right)=\frac{W_{i}-W_{0}}{W_{0}}\times 100$$

(3) $$Solubility\left(\%\right)=\frac{W_{i}-W_{f}}{W_{i}}\times 100$$

where *W*_0_ means the initial (dry) weight of the film, *W*_i_ means the weight after immersion, and *W*_f_ means the final dry weight after oven drying.

#### 4.8.5 Thermogravimetric Analysis

The thermal profile of the polymeric films, the most promising MFAq:LCHA association and chitosan were evaluated using simultaneous thermogravimetric analysis and differential thermal analysis (TGA/DTA) performed on a HITACHI STA‐7800 analyzer. Experiments were carried out under an air flow of 300 mL/min, with a heating rate of 30°C/min and a sensitivity of 1.0°C. Two mg of each sample were placed in open alumina crucibles for analysis. Data acquisition was performed using the TA7000 Measurement STA7200RV software. All analyses were conducted in triplicate, and data processing was carried out using Origin 9.0 software [70].

#### 4.8.6 Fourier‐transform Infrared Spectroscopy

To identify possible intermolecular interactions between the extracts components and chitosan, Fourier Transform Infrared Spectroscopy (FTIR) was performed using a Bruker Alpha FTIR spectrometer. The chitosan films containing MFAq:LCHA associations (MFAq:LCHA‐0.5%–2.5%) and the blank film (Film‐BL), and the MFAq and LCHA extracts were analyzed in attenuated total reflectance (ATR) mode using a PIKE ATR accessory coupled to the spectrometer. Spectra were recorded as the average of 64 consecutive scans, with a resolution of 4 cm^−1^, over a wavenumber range of 4000 to 400 cm^−1^. Spectral acquisition was carried out using the Perkin Elmer Spectrum Two software (Oxford Instruments), and data processing was performed using Origin 9.0 software.

#### 4.8.7 Mechanical Properties

Dynamic mechanical analysis of the films in axial and rotational modes was performed using an automated DHR‐1 rheometer (TA Instruments) equipped with a linear fixation DMA accessory, at a temperature of 25°C. For axial mode (tensile) testing, chitosan films containing MFAq:LCHA associations (MFAq:LCHA‐0.5%–2.5%) and blank film (Film‐BL) (3 × 0.4 cm) were mounted on the accessory and subjected to tensile deformation at a constant rate of 1000 µm/s until rupture. For rotational mode (shear) testing, the samples were fixed with a gap of 3 cm and subjected to rotation at an angular velocity of 1 rad/s. Experiments were conducted in quintuplicate, and stress versus gap (axial mode) and stress versus angle (rotational mode) curves were constructed for each sample. Data processing was carried out using TRIOS software and subsequently exported to GraphPad Prism 8.0.1.

### 4.9 Biological Evaluations

#### 4.9.1 Cell Culture Conditions

Peritoneal macrophages (primary cell line) were obtained from BALB/c mice (Protocol Number: 07/2018‐CEUA) (weighing 20–25 g) and cultured in RPMI‐1640 medium (Gibco), supplemented with 2 mM L‐glutamine, 10% fetal bovine serum (FBS), and 1% antibiotic solution (100 µg/mL penicillin and 100 µg/mL streptomycin). The procedure was accepted by the Committee on the Ethics of Animal Experiments of the Federal University of Juiz de Fora on May 10, 2018. The immortalized murine fibroblast cell line (L929‐ATCC CCL‐1 NCTC, RRID:CVCL_0462) was obtained from BCRJ (Rio de Janeiro Cell Bank) and subsequently cultured in DMEM (Dulbecco's Modified Eagle Medium) supplemented with 10% FBS and 1.0% antibiotic solution. Both cell lines were maintained in an incubator at 37°C with a 5.0% CO2 atmosphere.

#### 4.9.2 Cell Viability by MTT Assay

Peritoneal macrophages (2 × 10^5^ cells/well) were prepared at two different ways: seeding into 96‐well microplates for treatment with serial dilutions of MFAq:LCHA associations (18.75 up to 300 µg/mL); and seeding into 24‐well microplates for treatment with 0.2 × 0.2 cm of chitosan films containing MFAq:LCHA associations (MFAq:LCHA‐0.5%–2.5%) and blank film (Film‐BL) (equivalent to about 0.25 mg of extract in the film with the highest concentration). L929 fibroblasts (5 × 10^3^ cells/well) were also seeded into 24‐well microplates and exposed to film samples (0.2 × 0.2 cm). Both cell lines were incubated for 48 h (37°C, 5% CO_2_), cell viability was measured using the MTT assay [16]. Negative controls consisted of cells treated with 0.06% dimethyl sulfoxide (DMSO). The absorbance was measured at 570 nm (Thermo Scientific Multiskan GO, software 3.2). Cell viability was expressed as a percentage, determined by comparison with a negative control. Each concentration was tested in triplicate, and two independent experiments were conducted.

### 4.10 Anti‐Inflammatory Activity

#### 4.10.1 NO Production

The production of nitric oxide (NO) was indirectly evaluated by the Griess method [19]. Peritoneal macrophages (2 × 10^5^ cells/well) were incubated for 48 h (37°C, 5% CO2) with varying concentrations (18.75 up to 300 µg/mL) of the MFAq:LCHA associations and chitosan films containing MFAq:LCHA associations (MFAq:LCHA‐0.5%–2.5%) and blank film (Film‐BL) (0.2 × 0.2 cm, equivalent to about 0.25 mg of extract in the film with the highest concentration), and stimulated with 1 µg/mL LPS and 1 ng/mL IFN‐γ. The stimulation control consisted of stimulated but untreated macrophages, while the basal control consisted of unstimulated and untreated cells. The supernatant and Griess reagent were added to plates and incubated at room temperature for 10 min. The absorbance was measured at 540 nm using a UV‐Vis spectrophotometer (Thermo Scientific Multiskan GO, software 3.2). The NO concentration was determined by comparison with a standard curve prepared from serial dilutions of a sodium nitrite (NaNO_2_) solution. The IC50 (50% inhibitory concentration) results are presented as the mean ± standard deviation, expressed in µg/mL. Each concentration was tested in triplicate, and two independent experiments were conducted.

#### 4.10.2 Accumulation of Lipid Droplets (LDs)

The accumulation of lipid droplets (LDs) in peritoneal macrophages was evaluated following the method of Basselin et al. [], using the Nile Red marker (9‐diethylamino‐5H‐benzo[α]phenoxazine‐5‐one). Peritoneal macrophages (2 × 10^5^ cells/well) were incubated for 48 h (37°C, 5% CO_2_) with varying concentrations (75 up to 300 µg/mL) of the MFAq:LCHA associations and stimulated with 1 µg/mL LPS and 1 ng/mL IFN‐γ. The stimulation control consisted of stimulated but untreated macrophages, while the basal control consisted of unstimulated and untreated cells. Subsequently, the cells were washed with PBS and stained with 200 µL of Nile Red (10 µg/mL). Fluorescence was measured at 485 nm excitation and 528 nm emission (FLx800, BioTek Instruments, Inc., Winooski, VT, USA). Fluorescence intensity (A.U.) was used to express the results. Each concentration was tested in triplicate across two independent experiments.

#### 4.10.3 Pro‐Inflammatory Cytokines Production

The pro‐inflammatory cytokines IL‐6 (interleukin 6) and IL‐12 (interleukin 12) were quantified using a direct ELISA method, following the protocols provided in the commercial kits. Peritoneal macrophages (2 × 10^5^ cells/well) were incubated for 6 h (37°C, 5% CO_2_) with film samples (0.2 × 0.2 cm, equivalent to about 0.25 mg of extract in the film with the highest concentration) and the most promising MFAq:LCHA association, rate 1:1, (150 up to 300 µg/mL), stimulated with 1 µg/mL LPS and 1 ng/mL IFN‐γ for 24 h. The stimulation control consisted of stimulated but untreated macrophages, while the basal control consisted of unstimulated and untreated cells. The supernatant was collected, and the cytokines were quantified using a sandwich‐type direct ELISA method with the commercial BD OptEIATM kit (BD Biosciences), according to the manufacturer's protocol. Absorbance was measured at 450 nm using a ThermoScientific SkanIt Multiskan GO (software 3.2). Cytokine concentrations (pg/mL) were determined against a standard curve. The assay was performed in triplicate.

### 4.11 Antioxidant Activity

#### 4.11.1 DPPH Scavenging Method

DPPH radical scavenging activity was determined using the method described by Brand‐Williams et al. [24]. A solution of MFAq:LCHA associations and the positive control (rutin) was prepared at 1 mg/mL in methanol and subsequently diluted in a series. Chitosan films containing MFAq:LCHA associations (MFAq:LCHA‐0.5%–2.5%) and blank film (Film‐BL) of 0.4 × 0.4 cm (equivalent to about 1 mg of extract in the film with the highest concentration) were immersed in a methanol:water solution (9:1 v/v) and centrifuged at 2300 rpm for 10 min [73]. The supernatants were collected and used as stock solutions. The samples and a DPPH solution (20 µg/mL in methanol) were incubated for 30 min in a 96‐well microplate. The absorbance was measured at 517 nm (Thermo Scientific Multiskan GO, software 3.2). The IC50 (50% inhibitory concentration) was expressed as the mean ± standard deviation in µg/mL. Two independent experiments were conducted, each with triplicate measurements.

#### 4.11.2 β‐Carotene/Linoleic Acid System

Lipid peroxidation inhibition activity was evaluated using the method described by Melo and Mancini Filho [74]. In a 96‐well microplate, methanolic solutions of the MFAq:LCHA associations and positive control (rutin) were added with β‐carotene/linoleic acid emulsion (final concentration of 1.25 up to 40 µg/mL), followed by incubation at 45°C. The absorbance was measured at 470 nm every 15 min for 120 min (Thermo Scientific Multiskan GO, software 3.2). Two independent experiments were performed, each in triplicate. The oxidation curves were analyzed to calculate F1 (between 15 and 45 min) and F2 (between 75 and 90 min) using the tangent method. Results were expressed as the mean ± standard deviation of the IC50 in µg/mL and as the percentage of lipid peroxidation inhibition (%I):

(4) $$Inhibition\left(\%\right)=\frac{ABS_{Control}-ABS_{Sample}}{ABS_{Control}}\times 100$$

where ABS_Control_ means the absorbance of rutin; ABS_Sample_ means the absorbance of the samples.

#### 4.11.3 Total Antioxidant Capacity by Phosphomolybdenum Assay

Total antioxidant capacity (TAC) was evaluated using the phosphomolybdenum complex reduction method, as described by Prieto et al. [20]. Chitosan films containing MFAq:LCHA associations (MFAq:LCHA‐0.5%–2.5%) and blank film (Film‐BL) of 0.4 × 0.4 cm (equivalent to about 1 mg of extract in the film with the highest concentration) were immersed in a methanol:water solution (9:1 v/v) and centrifuged at 2300 rpm for 10 min; the supernatants were collected and used for the assay [15]. A stock solution of the positive controls (rutin and quercetin) was prepared at 0.5 mg/mL in methanol:water solution (9:1 v/v). The samples and the reagent solution were added to hermetically sealed test tubes, which were then incubated in a water bath. The absorbance was measured at 695 nm (Thermo Scientific Multiskan GO, software 3.2). Data were expressed as mean ± standard deviation of the percentage of relative antioxidant activity (RAA%) for rutin and quercetin following the equation:

(5) $$AAR\left(\%\right)=\frac{ABS_{Sample}-ABS_{BlankSample}}{ABS_{PC}-ABS_{BlankPC}}\times 100$$

where ABS_Sample_ means the absorbance of the sample; ABS_BlankSample_ means the absorbance of the sample without reagent addition; ABS_PC_ means the absorbance of the positive controls (quercetin or rutin); ABS_BlankPC_ means the absorbance of the controls without reagent.

### 4.12 Scratch Wound Healing Assay

Wound healing activity was evaluated using the scratch assay method proposed by Okur et al. [75]. L929 fibroblasts (5 × 10^3^ cells/well) were seeded into 24‐well microplates; after 24 h, a linear scratch was made in the adherent cell layer using a sterile pipette tip. Chitosan films containing MFAq:LCHA associations (MFAq:LCHA‐0.5‐2.5%) and blank film (Film‐BL) (0.1 × 0.1 cm, equivalent to about 0.06 mg of extract in the film with the highest concentration) and a solution of the most promising MFAq:LCHA association, rate 1:1, (concentration of 18.75 up to 37.50 µg/mL) were directly added to the cultures. As a control, cells were treated with only DMEM. Images were taken at 10× magnification using a microscope (FLx800, BioTek Instruments, Inc., Winooski, VT, USA) at 0, 24, and 48 h. To assess cell migration, the images were analyzed using ImageJ software (version 1.54). Two independent experiments were performed in triplicate, and cell migration rate (%) was calculated using the equation:

(6) $$Migrationrate\;\;\left(\%\right)=\frac{Area_{T0}-rea_{Tf}}{Area_{T0}}\times 100$$

where Area_T0_ means scratch area at 0 h; Area_Tf_ represents the scratch area at 24 or 48 h.

### 4.13 Statistical Analysis

All experiments were performed at least in triplicate, and the results are presented as mean ± standard deviation (SD). The sample size (n) for each experiment is indicated in the corresponding figure and table legends. Statistical analyses were carried out using GraphPad Prism 8.0.1 software (Erithacus Software Ltd). Comparisons among groups were performed using one‐way analysis of variance (ANOVA) followed by Bonferroni's post hoc test for multiple comparisons. Differences were considered statistically significant when *p* < 0.05.

Statistical details, including significance symbols and specific group comparisons, are described in the respective figure and table legends. Error bars are shown only for experiments involving replicated quantitative measurements and statistical analyses. Continuous instrumental profile analyses (e.g., FTIR, rheological curves, SEM, and thermal analyses) are presented as representative profiles and therefore do not include error bars. Rheological data and continuous curve analyses were processed using OriginPro 9.0 software (OriginLab Corporation, Northampton, MA, USA).

## Author Contributions

The study design, study mentorship, and manuscript critical editing were supplied by R.L.F., E.S.C., A.M.L.D., L.F.C.O., and G.C.M. The validation of the methodology, experimental development, and interpretation of results were conducted by P.L.P., J.B.F., M.R.C.C., L.A.C, N.S.M., A.S.O.L., and M.I.D. Each author authorized the completed version of the manuscript after contributing in part to its writing and editing.

## Funding

Grants were used to fund this effort from Conselho Nacional de Desenvolvimento Científico e Tecnológico (CNPq, Brazil—grant number: 408700/2021‐1) and Fundação de Amparo à Pesquisa do Estado de Minas Gerais (grant numbers: APQ‐01357‐21 and APQ‐04195‐25). Ari Sérgio de Oliveira Lemos were grant recipients of Coordenação de Aperfeiçoamento de Pessoal de Nível Superior (CAPES, Brazil). Lara Melo Campos, Elaine Soares Coimbra, and Rodrigo Luiz Fabri are grant recipients of CNPq. The article processing fee for publishing this research was paid by CAPES/Brazil (ROR identifier: 00 × 0ma614). For open access purposes, the authors have assigned the Creative Commons CC BY license to any accepted version of the article.

## Conflicts of Interest

The authors declare no conflicts of interest.

## Supporting information




**Supporting File 1**: cbdv71645‐sup‐0001‐SuppMat.pdf

## Data Availability

The publication contains all the data from this work. The license for the use of the plant species is registered with SISGEN/Brazil (A18AB08).
