# Supplementary material for: Synergistic Phytochemicals From Mitracarpus frigidus (Willd. ex Roem. & Schult.) K. Schum. and Lantana camara Linn. in Chitosan Biofilms: Antioxidant and Anti‐Inflammatory Activities With Wound‐Healing Potential
Source: Chem Biodivers. 2026 Sep 30;23(10):e71645. doi: 10.1002/cbdv.71645 (PMC13627852; doi:10.1002/cbdv.71645)

**Supplementary Figure S1.** UFLC-QTOF-MS chromatographic profile of the MFAq:LCHA 1:1 association with annotation of the major identified metabolites. Numbered peaks correspond to compounds identified by mass spectrometric analysis. Chromatogram for data in Table 3.

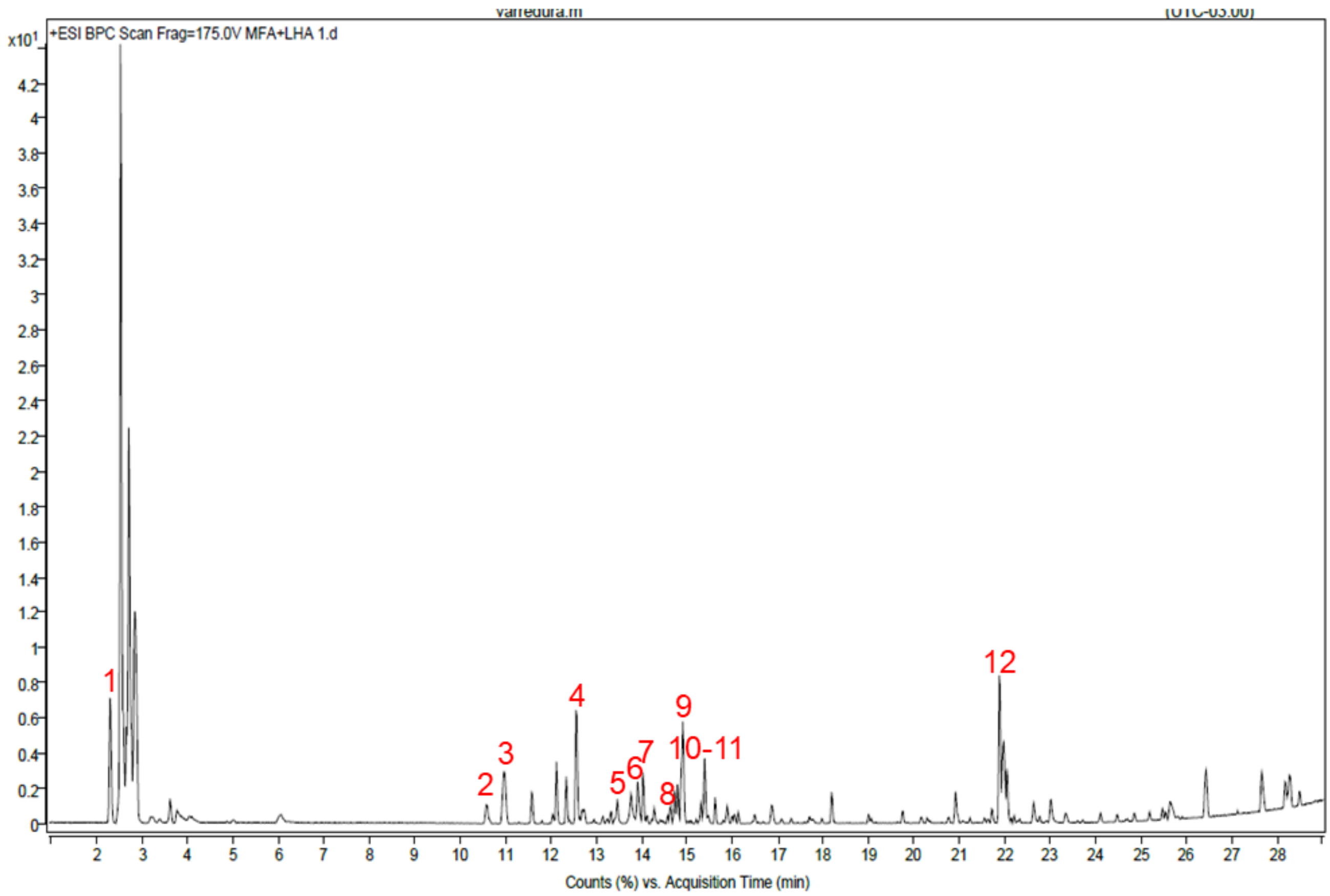

Supplement: Supplementary file 1 [file CBDV-23-e71645-s001.pdf]
